# Gut Microbiome Dysbiosis in Metabolic Syndrome: Current Evidence and Emerging Perspectives

**DOI:** 10.3390/nu18101540

**Published:** 2026-05-13

**Authors:** Fatma Shehata, Karen M. Dwyer, Sean L. McGee, Leni R. Rivera

**Affiliations:** 1 School of Medicine, Institute for Mental and Physical Health and Clinical Translation, Deakin University, Geelong, VIC 3220, Australia; s222396088@deakin.edu.au (F.S.); sean.mcgee@deakin.edu.au (S.L.M.); 2 The Royal Melbourne Hospital, The University of Melbourne, Parkville, VIC 3050, Australia; karen.dwyer@mh.org.au

**Keywords:** gut microbiota, metabolic syndrome, dysbiosis, prebiotics, probiotics, synbiotics, postbiotics, fecal microbiota transplantation

## Abstract

The gut microbiota plays a crucial role in human metabolism, and disruptions to its composition, particularly reductions in bacterial diversity, have been increasingly associated with the development of metabolic syndrome (MetS). MetS encompasses a constellation of interrelated metabolic risk factors, including central obesity, insulin resistance, dyslipidemia, and hypertension, which collectively elevate the risk of cardiovascular and cerebrovascular disease. A comprehensive understanding of the mechanisms underlying MetS is therefore critical for the development of effective preventive and therapeutic strategies. Complex interactions between the gut microbiota and host metabolic pathways are mediated by multiple factors, including microbial metabolites, inflammatory signaling, and host immune responses. This narrative review characterizes the clinical manifestations of MetS and alterations in gut microbiota composition, characterized by an overrepresentation of potentially pathogenic taxa and a concomitant decline in beneficial microbial species. In addition, we discuss current and emerging approaches to microbiota modulation, including prebiotics, probiotics, synbiotics, postbiotics, and fecal microbiota transplantation, and evaluate their potential roles in the prevention and management of MetS. We identify critical evidence gaps and propose research priorities for evidence-based clinical strategies for MetS management and prevention.

## 1. Introduction

The prevalence of metabolic syndrome (MetS) has risen markedly over recent decades, emerging as a major public health challenge that affects individuals across diverse age groups and populations. This increase closely parallels the widespread adoption of Westernized diets and sedentary lifestyles. MetS is characterized by a cluster of interrelated risk factors, including central obesity, hyperglycemia, dyslipidemia, hypertension, and hyperuricemia, and is strongly associated with the development of chronic diseases such as type 2 diabetes (T2D) and cardiovascular disease.

The pathogenesis of MetS is multifactorial and involves complex, interdependent mechanisms, including insulin resistance, chronic low-grade inflammation, autonomic dysfunction, and enhanced oxidative stress [1,2]. In recent years, mounting evidence has implicated gut microbiota dysbiosis as an additional and potentially modifiable risk factor contributing to MetS development and progression [3]. The gut microbiota comprises a highly diverse and dynamic community of microorganisms that reside within the gastrointestinal tract and maintain a symbiotic relationship with the human host [4]. It plays a crucial role in host metabolic homeostasis by facilitating the fermentation of indigestible dietary components, synthesizing essential vitamins, protecting against pathogenic colonization, and supporting immune system function [5].

Dysbiotic patterns, characterized by reduced microbial diversity and an imbalance between beneficial and pathogenic taxa, have been associated with key features of MetS, including insulin resistance, systemic inflammation, and abnormal lipid metabolism [3]. These dysbiotic changes may influence host physiology through multiple mechanisms, such as altered production of short-chain fatty acids (SCFAs), disruption of bile acid metabolism, increased intestinal permeability, and activation of pro-inflammatory pathways [6,7].

Given the central role of the gut microbiota in metabolic regulation, microbiota-targeted therapeutic strategies have emerged as promising targets. Interventions such as probiotics, prebiotics, synbiotics, postbiotics, and fecal microbiota transplantation (FMT) represent promising approaches for restoring microbial balance and improving metabolic outcomes. Accordingly, this narrative review aims to synthesize current evidence on the relationship between gut microbiota dysbiosis and the individual components of MetS, and to critically evaluate the potential of gut microbiota-targeted therapies in the prevention and management of this increasingly prevalent metabolic disorder.

## 2. Literature Search Strategy

This narrative review was conducted through a structured literature search to identify studies examining the relationship between gut microbiota and MetS. A structured search of the NCBI PubMed database was performed for studies published between January 2001 and February 2026. The search strategy used combinations of keywords, including “gut microbiota”, “metabolic syndrome”, “dysbiosis”, “insulin resistance”, “obesity”, “inflammation”, “prebiotics”, “probiotics”, “synbiotics”, “postbiotics”, and “fecal microbiota transplantation”. Both human and animal studies were considered to provide a comprehensive overview of mechanistic and clinical evidence. Studies reporting gut microbial composition in MetS were included regardless of sequencing platform (16S rRNA gene sequencing or shotgun metagenomics) or study setting.

Search results were exported and duplicates removed. Titles and abstracts were screened for relevance, followed by full-text assessment based on predefined criteria, including relevance to MetS, availability of gut microbiota data, and appropriate experimental or observational design. Studies were excluded if they did not assess gut microbiota composition, were not relevant to MetS or related metabolic phenotypes, or lacked primary microbiome data. Reference lists of included studies were also screened for additional relevant publications. A narrative synthesis approach was used due to heterogeneity across studies. Findings were interpreted cautiously, distinguishing between associative and causal relationships and avoiding over-extrapolation.

## 3. Dysbiosis and Metabolic Dysfunction

The gut microbiota is continually exposed to multiple classes of stressors associated with modern lifestyles. Dietary factors include Western diets (high in processed foods, saturated fats and low in fiber) and additives (e.g., emulsifiers and sweeteners), while environmental factors include antibiotics, other pharmaceuticals, pesticides, heavy metals, persistent organic pollutants, and mycotoxins [8,9,10,11,12,13,14]. Chronic exposure to these stressors can alter microbial composition and metabolic activity, resulting in gut dysbiosis with detrimental effects on host health [15].

Gut dysbiosis has been associated with a wide spectrum of chronic conditions, including gastrointestinal disorders (inflammatory bowel disease and irritable bowel syndrome) [16,17], central nervous system diseases (Alzheimer’s and Parkinson’s diseases) [18,19], immune-mediated conditions (allergy diseases and rheumatoid arthritis) [20,21], and metabolic disorders (obesity and T2D) [22,23].

At the compositional level, dysbiosis typically involves a reduced abundance of key beneficial taxa (particularly butyrate-producing bacteria) alongside an increased presence of potentially pathogenic or pro-inflammatory taxa [24]. Reduced microbial diversity and alterations in the Firmicutes:Bacteroidetes (F:B) ratio have frequently been associated with obesity, T2D, and impaired glycemic control [25]. However, the clinical utility of the F:B ratio as a dysbiosis marker remains controversial, as considerable heterogeneity in this ratio exists across populations and disease phenotypes [26]. More importantly, dysbiosis involves loss of microbial metabolic function (particularly SCFA-producing capacity) rather than specific compositional signatures alone [21,27]. Dysbiosis also substantially impacts host immune regulation. Cytokine production, particularly pro-inflammatory mediators such as tumor necrosis factor (TNF) and interferons (IFNs), can be influenced by microbial metabolic pathways [28]. Dysbiotic microbial profiles have been linked to aberrant cytokine responses and impaired immune homeostasis, contributing to chronic inflammation and metabolic dysfunction—key features of MetS [29,30,31].

Collectively, these environmental and dietary stressors disrupt microbial ecological balance by reducing beneficial commensals and promoting opportunistic and pro-inflammatory taxa. This is accompanied by early functional impairments, including reduced SCFA production and weakened intestinal barrier support, which together promote low-grade immune activation. These early alterations provide a mechanistic link between environmental exposures, gut dysbiosis, and the initiation of metabolic disturbances.

The following section focuses on how these dysbiotic alterations are specifically observed in MetS and how they contribute to its pathophysiology.

## 4. Gut Microbiota Alterations in Metabolic Syndrome

While gut microbiota alterations in individual metabolic disorders (obesity, T2D, and hypertension) have been extensively characterized, fewer studies have examined dysbiosis in the context of MetS as an integrated metabolic condition. However, accumulating evidence indicates that specific microbial perturbations contribute significantly to MetS pathophysiology (Table 1). Given the gut microbiota’s central role in maintaining host metabolic homeostasis, dysbiosis induced by dietary, environmental, and lifestyle factors can trigger physiological disturbances that increase susceptibility to MetS [32].

### 4.1. Reduced Microbial Diversity and Global Compositional Shifts in Adults with MetS

One of the most consistent findings is a reduction in gut microbial diversity among individuals with MetS compared with metabolically healthy controls [3,33]. This loss of diversity is typically accompanied by compositional shifts characterized by a reduction in key beneficial taxa such as *Akkermansia muciniphila* and *Bifidobacterium* spp., and an overrepresentation of pro-inflammatory or endotoxin-producing bacteria, including *Desulfovibrio* and members of the Enterobacteriaceae family. Such alterations disrupt metabolic and immune signaling pathways, exacerbating immune dysregulation and promoting chronic low-grade systemic inflammation [34,35,36].

### 4.2. Loss of Protective Taxa in Adults with MetS: SCFA-Producing and Barrier-Associated Bacteria

A hallmark of dysbiosis in MetS is the substantial depletion of microbial taxa essential for metabolic health and intestinal barrier maintenance [37]. Several studies have highlighted significant reductions in key short-chain fatty acid (SCFA)-producing species, including *Faecalibacterium prausnitzii*, *Roseburia intestinalis*, and *Anaerostipes* spp., in individuals with MetS compared with healthy controls [3,38,39]. Similarly, *Akkermansia muciniphila* (a mucus-associated bacterium crucial for barrier integrity) is consistently reduced in MetS populations. Notably, *Akkermansia muciniphila* abundance is inversely correlated with MetS diagnosis, with higher levels of this bacterium associated with lower odds of adverse metabolic traits, including increased body mass index, elevated low-density lipoprotein cholesterol, hypertriglyceridemia, hyperglycemia, and hyperinsulinemia [40,41]. Moreover, lower abundances of other SCFA producers such as *Alistipes*, *Bifidobacterium*, and *Lactobacillus* have been significantly correlated with elevated fasting glucose and adiposity measures, further linking SCFA deficiency to MetS pathophysiology [3,38].

**Table 1:** Key microbial taxa depleted in MetS and their metabolic associations.

Taxon	Functional Role	Observed Change in MetS	Associated Metabolic Impact	References
*Faecalibacterium prausnitzii*	Major SCFA (butyrate) producer; anti-inflammatory; supports gut barrier	↓ Reduced	Linked to impaired barrier integrity and metabolic dysfunction	[3,38,39]
*Roseburia intestinalis*	Butyrate producer; maintains epithelial health	↓ Reduced	Associated with reduced SCFA levels and metabolic imbalance	[3,38,39]
*Anaerostipes* spp.	SCFA (butyrate) production	↓ Reduced	Contributes to SCFA deficiency in MetS	[3,38,39]
*Akkermansia muciniphila*	Mucus-degrading; maintains gut barrier integrity	↓ Reduced	Inversely associated with BMI, LDL, triglycerides, glucose, and insulin levels	[40,41]
*Alistipes*	SCFA production; metabolic regulation	↓ Reduced	Correlated with increased fasting glucose and adiposity	[3,38]
*Bifidobacterium*	SCFA production; gut barrier support; anti-inflammatory	↓ Reduced	Associated with elevated fasting glucose and adiposity	[3,38]
*Lactobacillus*	SCFA production; immune modulation; barrier support	↓ Reduced	Linked to increased adiposity and metabolic dysregulation	[3,38]

↓ = decrease.

#### Mechanistic Consequence: SCFA Deficiency and Barrier Dysfunction

A critical consequence of depleted SCFA-producing bacteria is the reduced production of SCFAs, particularly butyrate, which plays a crucial role in maintaining intestinal barrier integrity and insulin sensitivity. Butyrate promotes epithelial barrier function through specific molecular mechanisms: it binds to G-protein-coupled receptors such as GPR109A, which has been shown in mouse models to enhance tight junction protein expression and protect barrier integrity in a receptor-dependent manner, preventing pathogen translocation and inflammation [42]. Additionally, butyrate inhibits histone deacetylase (HDAC) activity by binding to the catalytic Zn^2+^ site within the enzyme pocket, thereby inhibiting class I HDACs (HDAC1, HDAC2, HDAC3, and HDAC8). This inhibition increases the expression of genes involved in epithelial function and tight junction stability in colitis models [43,44,45,46]. Collectively, these actions regulate immune signaling and support epithelial homeostasis, actions that enhance mucus production and barrier-protective immune responses, and metabolic support for intestinal epithelial cells, which rely heavily on butyrate as an energy source [47].

Decreased SCFA availability compromises these protective mechanisms, leading to progressive deterioration of epithelial tight junctions through reduced claudin and occludin expression [48,49,50]. This facilitation of increased intestinal permeability allows lipopolysaccharide (LPS) and other microbial pathogen-associated molecular patterns (PAMPs) derived from Gram-negative bacteria to translocate into the systemic circulation [51,52]. Elevated circulating LPS concentrations trigger potent inflammatory cascades, including activation of toll-like receptor 4 (TLR4) signaling, leading to the production of pro-inflammatory cytokines such as TNF-α, IL-6 and IL-17, thereby promoting systemic inflammation [53,54,55]. Mechanistically, TLR4 activation induces MyD88-dependent NF-κB signaling and subsequent activation of IκB kinase-β (IKK-β), which promotes serine phosphorylation of insulin receptor substrate-1 (IRS-1), impairing downstream insulin signaling and contributing to insulin resistance [56,57]. In the liver, chronic endotoxemia enhances TLR4-mediated activation of sterol regulatory element-binding protein-1c (SREBP-1c), increasing de novo lipogenesis while suppressing fatty acid oxidation, thereby promoting triglyceride accumulation and hepatic steatosis [58]. Systemic inflammation also disrupts apolipoprotein metabolism and very-low-density lipoprotein handling, contributing to dyslipidemia [59,60]. Furthermore, LPS-induced TLR4 signaling promotes endothelial dysfunction by increasing oxidative stress and reducing nitric oxide bioavailability, accelerating atherosclerotic plaque development and increasing cardiovascular risk [61,62].

Collectively, this gut barrier–endotoxemia–TLR4 inflammatory axis provides a mechanistic link between microbiome-derived metabolic dysfunction and the cardinal features of MetS [63].

### 4.3. Enrichment of Pro-Inflammatory and Endotoxin-Producing Taxa

Enrichment of Proteobacteria, particularly LPS-producing genera such as Enterobacteriaceae and *Escherichia–Shigella*, has been consistently reported in individuals with MetS [64]. This increase in pro-inflammatory bacteria contributes to metabolic endotoxemia. Elevated plasma LPS concentrations have been correlated with increases in these taxa and have been implicated not only in MetS components (hypertension, dyslipidemia, and insulin resistance) but also in more severe cardiometabolic complications, including preeclampsia, highlighting overlapping microbial pathways across cardiometabolic disorders [65,66,67].

Beyond Proteobacteria, several additional taxa are consistently enriched in MetS populations with pro-inflammatory consequences. *Ruminococcus gnavus*, a species previously associated with inflammatory bowel disease, is increased in MetS cohorts and correlates with systemic inflammatory markers [68,69].

Additional taxa enriched in MetS include Peptostreptococcaceae, which has been positively associated with increased BMI and insulin resistance [70], as well as *Prevotella*, a genus with context-dependent metabolic effects [71,72,73,74]. While Prevotella is generally linked to metabolic disorders and systemic inflammation [75], emerging evidence suggests species-specific phenotypes. For example, *Prevotella copri* has been associated with increased branched-chain amino acid production and insulin resistance [76], yet other *Prevotella* species show variable associations depending on diet and host factors [74,77,78,79]. Conversely, other studies in animal models demonstrate that *P. copri* supplementation can improve glucose homeostasis, enhance intestinal gluconeogenesis via succinate production, and increase GLP-1 secretion, leading to improved glucose tolerance and insulin sensitivity [73,80,81,82]. Notably, elevated levels of *Gemella sanguinis* and *Eubacterium siraeum*, bacteria associated with systemic infections and autoimmune conditions, have also been reported in MetS populations, suggesting that dysbiosis in MetS extends beyond recognized metabolic pathogens to include pathobionts typically controlled by healthy immune homeostasis [3,83,84].

Recent evidence has identified additional pro-inflammatory compositional shifts, including increased abundance of *Anaerotignum lactatifermentans* [85], which may be harmful, as this species has previously been shown to be enriched in individuals with elevated glucose levels and higher BMI [86]. Conversely, decreased abundance of *Megasphaera* (butyrate-producing genus with established beneficial metabolic effects) represents an additional mechanism of functional SCFA deficit in MetS [85,87].

## 5. Dysbiosis Signatures Across Pediatric Populations and Models

While the dysbiotic patterns described above represent a core dysbiotic phenotype in MetS, important variations exist across population groups, age categories, and experimental models. Understanding these variations provides mechanistic insight into MetS heterogeneity and informs the tailoring of dysbiosis-targeted interventions.

### 5.1. Distinct Dysbiotic Patterns in Pediatric Populations and Early-Life Programming

Dysbiosis in childhood obesity associated with MetS exhibits distinct patterns and timing compared with adult populations, with implications for metabolic programming and disease prevention [88,89,90]. In pediatric populations, Gallardo-Becerra et al. demonstrated that children with obesity associated with MetS exhibited increased abundances of Firmicutes and Proteobacteria, along with specific taxa, including Coriobacteriaceae, *Clostridia*, *Coprococcus*, and *Catenibacterium*, which have been positively correlated with triglyceride levels, BMI, and waist circumference. In contrast, reductions in Bacteroidetes and *Parabacteroides distasonis* in children were negatively associated with triglycerides and BMI, highlighting early-life microbial signatures linked to metabolic dysfunction [91]. Similarly, Xiao et al. reported enrichment of Erysipelotrichaceae, Pasteurellaceae, Enterobacteriaceae, and Pseudomonadaceae in individuals with MetS, alongside increased abundance of *Escherichia*, *Enterococcus*, and *Turicibacter* [92]. These taxa include several LPS-producing bacteria known to activate pro-inflammatory immune responses and contribute to insulin resistance, atherosclerosis, and cardiovascular disease [64,93].

The clinical significance of pediatric dysbiosis relates to the concept of metabolic programming and the hypothesis that microbial dysbiosis during critical developmental windows in infancy and childhood establishes trajectories of metabolic dysfunction that persist or amplify through adulthood [94,95,96]. Early-life dysbiosis may fundamentally alter intestinal immune development, barrier maturation, and metabolic substrate utilization in ways that predispose to lifelong metabolic abnormalities [97,98]. Understanding whether dysbiosis in childhood MetS represents a reversible state or an established developmental trajectory has important implications for the timing and intensity of dysbiosis-targeted interventions.

### 5.2. Animal Models: Mechanistic Insights into Dysbiosis and MetS

While human cohort studies provide robust associative evidence linking dysbiosis to MetS, animal models have been instrumental in establishing causality and elucidating mechanistic pathways. Mouse models of diet-induced obesity and MetS have consistently demonstrated that dysbiosis precedes or accompanies metabolic abnormalities and, critically, that transfer of dysbiotic microbiota from MetS animals to germ-free recipients is sufficient to transfer MetS like metabolic abnormalities, supporting a potential contributory role for gut dysbiosis in MetS development.

In a MetS mouse model, Suriano et al. observed an increase in *Romboutsia*, which positively correlated with obesity, adipocyte hypertrophy, fat mass, and impaired glucose metabolism [99]. Conversely, *Parasutterella*—a taxon negatively associated with obesity and inflammatory markers—was significantly reduced, suggesting a potential protective role [99].

Similarly, Chen et al. reported higher abundances of multiple taxa (*Lactococcus*, *Streptococcus*, *Roseburia*, *Romboutsia*, *Clostridium_XVIII*, *Christensenella*, and *Staphylococcus*) in MetS mouse models, with many of these genera previously linked to high-fat diets, metabolic inflammation, and obesity [100,101,102]. In contrast, beneficial taxa such as *Parabacteroides*, *Clostridium sensu stricto*, and *Anaerofustis* were depleted [103].

Another mechanistic study using a T2D-induced MetS model found reduced Bacteroidetes, with dominance of *Lachnospiraceae*, *Muribaculaceae*, *Escherichia–Shigella*, and *Lachnoclostridium*, taxa associated with impaired glucose metabolism and increased inflammation [104]. Importantly, metabolic interventions (dietary, pharmaceutical, or microbiota-modulating) that ameliorated MetS phenotypes in these models were accompanied by restoration of dysbiotic profiles resembling those of healthy controls, providing evidence that dysbiosis reversal is mechanistically linked to metabolic improvement [105,106,107].

Collectively, evidence from animal models suggests that dysbiosis does not merely accompany MetS but rather is a causally contributing factor whose reversal can therapeutically improve MetS pathologies.

## 6. Common Microbial Signatures Across Metabolic Diseases

**Figure 1:**
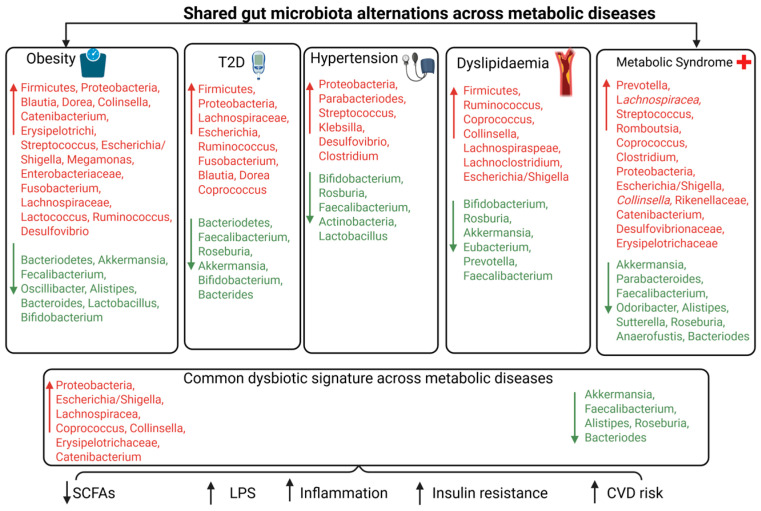
Shared gut microbiota alterations across metabolic diseases contributing to metabolic syndrome. Obesity, type 2 diabetes (T2D), hypertension, and dyslipidemia exhibit overlapping gut microbial alterations, characterized by enrichment of pro-inflammatory and LPS-producing taxa (e.g., Proteobacteria and *Escherichia/Shigella*) and depletion of beneficial SCFA-producing or mucus-associated bacteria (e.g., *Akkermansia* and *Faecalibacterium*). These shared dysbiotic features contribute to metabolic syndrome through reduced SCFA production, metabolic endotoxemia, low-grade inflammation, insulin resistance, and increased cardiovascular risk. Created in BioRender. Shehata, F. (2026) https://BioRender.com/z450qe0.

Although discrepancies exist across studies due to methodological differences, including variation in DNA extraction protocols that bias recovery of specific taxa (e.g., differential lysis of Gram-positive bacteria) [108], selection of different 16S rRNA gene hypervariable regions (e.g., V3–V4 vs. V4–V5) [109], use of distinct taxonomic reference databases (e.g., SILVA vs. Greengenes) [110], differences in sequencing depth [111], and alternative bioinformatic pipelines (OTU vs. ASV) [112], as well as population heterogeneity and disease complexity [113,114], no single taxon has been identified as solely responsible for MetS. Instead, overlapping microbial patterns are observed across metabolic conditions, including obesity, T2D, hypertension, and dyslipidemia (Figure 1). Notably, increased abundances of Firmicutes, Proteobacteria, Lachnospiraceae, Erysipelotrichaceae, *Escherichia/Shigella*, *Coprococcus*, *Collinsella*, and *Catenibacterium* are frequently reported across these conditions [64,91].

Conversely, reductions in *Akkermansia*, *Bacteroides*, *Faecalibacterium*, *Roseburia*, *Alistipes*, *Bifidobacterium*, and *Lactobacillus* are frequently associated with MetS and related metabolic disorders [3,38,99]. These shared microbial signatures suggest a convergent dysbiotic phenotype associated with MetS and its individual clinical components (Figure 1). Combined with mechanistic evidence from animal models, this establishes dysbiosis as a modifiable risk factor and therapeutic target in MetS.

## 7. Gut Microbiota-Targeted Interventions

**Figure 2:**
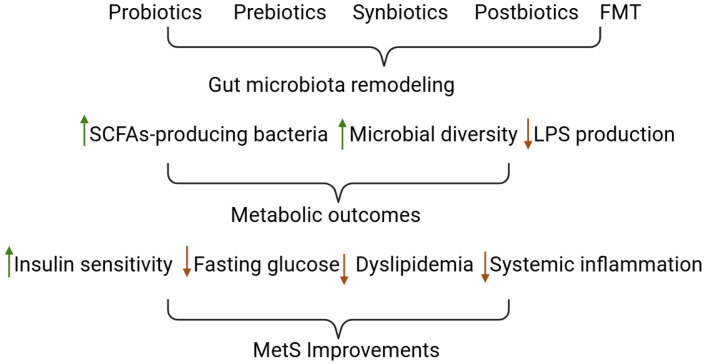
Conceptual workflow of gut microbiota-targeted interventions in metabolic syndrome. This figure illustrates a summary of gut microbiota-targeted interventions—including probiotics, prebiotics, synbiotics, postbiotics and fecal microbiota transplantation (FMT)—and their associated metabolic outcomes in MetS. These interventions are shown to promote gut microbiota remodeling, characterized by increased abundance of short-chain fatty acid (SCFA)-producing bacteria, enhanced microbial diversity, and reduced lipopolysaccharide (LPS) production. These microbial shifts are associated with improvements in key metabolic parameters, including enhanced insulin sensitivity, reduced fasting glucose levels, improved lipid profiles, and decreased systemic inflammation, collectively contributing to overall improvement in MetS features. Created in BioRender. Shehata, F. (2026) https://BioRender.com/s261oqw.

The dysbiosis observed in MetS, characterized by reduced SCFA production, increased LPS burden, and depletion of key barrier-supporting taxa, represents a potentially modifiable risk factor. Consequently, therapeutic strategies aimed at restoring gut microbial balance have emerged as promising approaches for the prevention and management of MetS (Table 2, Table 3 and Table 4; Figure 2).

Gut microbiota-targeted interventions, including probiotics, prebiotics, synbiotics, postbiotics, and FMT, aim to reverse dysbiosis by selectively enriching beneficial microbial taxa, enhancing microbial metabolite production, and attenuating inflammatory signaling pathways. Notably, although a substantial number of studies have investigated these interventions in obesity and MetS, relatively few have comprehensively characterized associated changes in gut microbiota composition, with most primarily focusing on metabolic outcomes. While preclinical and clinical evidence support the metabolic benefits of such interventions, further studies are required to clarify their efficacy, durability, and mechanisms of action in individuals with MetS [115].

### 7.1. Probiotics

Probiotics are defined as “live microorganisms that, when administered in adequate amounts, confer a health benefit on the host” [116]. They represent a microbiota-targeted intervention strategy aimed at enriching potentially beneficial bacteria while potentially suppressing dysbiotic taxa. Probiotics exert health effects through multiple mechanisms, including modulation of immune responses, production of antimicrobial compounds, enhancement of intestinal barrier function, and functional interactions with the resident gut microbiota that may reshape overall community composition [117,118]. These multiple mechanisms establish probiotics as potentially effective microbiota modulating tools, particularly in settings where conventional treatments are limited, ineffective, or costly [119].

Commonly used probiotic microorganisms include bacterial strains from the genera *Lactobacillus*, *Bifidobacterium*, *Lactococcus*, *Streptococcus*, and *Enterococcus*. In addition, selected Gram-positive *Bacillus* species and yeast strains such as *Saccharomyces* spp. are incorporated into probiotic formulations due to their stability and demonstrated health benefits [120]. More recently, the mucin-degrading bacterium *Akkermansia muciniphila* has emerged as a promising next-generation probiotic candidate due to its unique ability to strengthen gut barrier integrity, modulate host metabolism and immune function, and improve metabolic health outcomes [121,122]. Both human and animal studies report inverse associations between *A. muciniphila* abundance and obesity, T2D, systemic inflammation, and other metabolic disorders, suggesting a protective role against cardiometabolic dysfunction [123].

Preclinical evidence demonstrates that specific probiotic strains can reshape dysbiotic microbial communities and mitigate features of MetS. In a representative study, Wang et al. investigated the effects of *Lactobacillus paracasei* CNCM I 4270 and *Bifidobacterium animalis* subsp. lactis I 2494 in C57BL/6J mice fed a high-fat diet (60% fat) for 12 weeks [124]. Supplementation with both strains significantly reduced body weight gain without altering food intake, lowered blood glucose levels, improved glucose–insulin homeostasis and protected against diet-induced hepatic steatosis [124].

At the microbiota level, probiotic administration attenuated the high-fat diet-induced expansion of Proteobacteria and prevented reductions in Actinobacteria [124]. Beneficial taxa enrichment included *Bifidobacterium*, *Olsenella*, *Barnesiella*, *Allobaculum*, *Butyrivibrio*, and members of the Lachnospiraceae family—taxa that are negatively correlated with MetS phenotypes. Conversely, taxa positively associated with metabolic dysfunction (*Alistipes*, *Desulfovibrionaceae*, *Oscillibacter*, *Clostridium*, and *Dorea*) were significantly reduced. Notably, *Bifidobacterium* supplementation reduced adipocyte size and hepatic TNF α expression and tended to lower circulating lipopolysaccharide load (measured as serum LBP), suggesting reduced metabolic endotoxemia. However, leptin gene expression remained unchanged, suggesting that metabolic improvements occurred through pathways distinct from leptin-mediated energy homeostasis [124]. Mechanistically, these compositional shifts were accompanied by increased acetate production—a major SCFA—with restoration of disrupted microbial fermentation pathways [124]. Acetate can influence systemic inflammation and metabolic homeostasis through multiple pathways. In rodent models, acetate attenuates NLRP3 inflammasome activation in macrophages and reduces inflammatory signaling via GPR43/FFAR2-mediated mechanisms, protecting against endotoxemia and inflammatory responses [125,126,127]. Additionally, acetate has been shown to suppress histone deacetylase activity in cardiac tissue in diabetic and metabolic disease models, which is associated with improvements in lipid metabolism, oxidative stress, and tissue integrity [126]. Additional preclinical studies demonstrate the metabolic benefits of *Bifidobacterium* supplementation in high-fat diet-induced models of MetS [128]. A high-fat diet-induced rat model showed that *Bifidobacterium longum* supplementation restored intestinal bifidobacterial abundance and ameliorated metabolic dysfunction. Compared with untreated high-fat diet controls, *B. longum* reduced body weight gain, adiposity, fasting glucose, triglycerides, and systolic blood pressure whilst improving insulin sensitivity [128]. These were accompanied by reduced plasma lipopolysaccharide and interleukin-1β, alongside decreased intestinal inflammatory markers, demonstrating attenuation of metabolic endotoxemia and inflammation. Notably, *B. longum* upregulated intestinal Regenerating I gene expression, suggesting enhanced gut barrier repair and growth factor signaling [128].

Similarly, *Bifidobacterium adolescentis* supplementation in high-fat diet rats over 12 weeks reversed diet-induced metabolic disturbances. High-fat feeding reduced intestinal Bifidobacterium levels and induced visceral fat accumulation, hepatic steatosis, reduced muscle mass, pancreatic atrophy, and impaired insulin sensitivity [129]. *B. adolescentis* supplementation restored gut *Bifidobacterium* abundance and partially or fully reversed these alterations, showing reduced visceral fat and hepatic steatosis, improved insulin sensitivity (glucose infusion rate), and prevented pancreatic weight loss [129]. Collectively, these preclinical findings demonstrate that specific probiotic strains can beneficially reshape functionally relevant gut microbial communities and improve MetS-associated metabolic parameters in animal models.

Human clinical trials extend these findings, demonstrating that probiotic interventions enrich metabolically beneficial taxa across diverse strain formulations and populations. Tenorio Jiménez et al. demonstrated that daily intake of a probiotic containing *Lactobacillus reuteri* V3401 for 12 weeks in adults with MetS led to significant reductions in inflammatory biomarkers, including interleukin 6 (IL 6) and soluble vascular cell adhesion molecule 1 (sVCAM 1), compared with placebo [130]. These reductions indicate decreased systemic inflammation, a key driver of MetS pathophysiology. Additionally, the intervention increased the abundance of *Verrucomicrobia* and *Akkermansia*, microbial taxa consistently associated with improved metabolic health, including reductions in metabolic endotoxemia, adipose tissue inflammation, and insulin resistance [131,132,133]. However, no significant changes were observed in the HOMA IR index, indicating that metabolic improvements may occur independently of measurable changes in insulin resistance over the study duration.

Similarly, more recent evidence from Xiao et al. reported that an 11-week probiotic intervention using *Bifidobacterium adolescentis* CCFM8630 and *Lactobacillus reuteri* CCFM8631 in individuals with MetS significantly altered gut microbiota composition. An increased abundance of Erysipelotrichaceae, Fusobacteriaceae, Alcaligenaceae, Pasteurellaceae, and Enterobacteriaceae was observed following supplementation [92]. At the genus level, *Fusobacterium*, *Phascolarctobacterium*, *Peptostreptococcus*, *Bacteroides*, and *Lachnospira* were significantly enriched. Importantly, *Phascolarctobacterium* abundance was negatively correlated with fasting blood glucose, fasting insulin, and hip circumference, while *Lachnospira* and *Bacteroides* abundances were inversely associated with fasting blood glucose levels, suggesting potential links between probiotic-induced microbial shifts and metabolic parameter improvements [92].

Clinical trials focusing on *Bifidobacterium* species demonstrate consistent metabolic and inflammatory benefits. A 45-day intervention in individuals with MetS receiving fermented milk containing *B. lactis* HN019 significantly reduced body mass index, total and LDL cholesterol, and pro-inflammatory cytokines (TNF-α and IL-6) compared with controls [134]. A separate 90-day double-blind study in individuals with MetS showed further improvements with reduced interleukin-6, homocysteine, and hydroperoxide levels alongside increased adiponectin and nitric oxide metabolites, indicating improvements in systemic inflammation, oxidative stress, and endothelial function [135]. Collectively, these findings suggest that *B. lactis* HN019 exerts cardiometabolic benefits in a MetS-controlled trial in 45 individuals with MetS in which *Bifidobacterium breve* strains BR03 and B632 were evaluated over 3 months. Supplementation significantly improved metabolic outcomes, demonstrating reduced BMI, waist circumference, visceral fat ratio, fasting glucose, HbA1c, total cholesterol, triglycerides, and LDL cholesterol, as well as increased HDL cholesterol [136]. These improvements likely reflect enhanced SCFA production and improved insulin sensitivity, supporting *B. breve* as a beneficial adjunct for MetS management and supporting the anti-inflammatory mechanisms observed in preclinical models [136].

Collectively, preclinical and clinical evidence demonstrate that probiotics, particularly *Bifidobacterium* and *Lactobacillus* species, can beneficially modulate gut microbiota composition, attenuate systemic inflammation, improve gut barrier integrity, and modestly improve cardiometabolic parameters in MetS. However, despite consistent enrichment of beneficial taxa such as *Akkermansia*, *Lachnospira*, and *Bacteroides*, improvements in core MetS diagnostic features remain variable across human studies. This discrepancy between microbial shifts and metabolic outcomes reflects several factors, including heterogeneity in strain composition, baseline microbiota diversity, individual colonization success, limited intervention duration (8–12 weeks), and potentially the requirement for longer periods to manifest clinically meaningful improvements. Notably, these findings suggest that microbiota-mediated mechanisms alone may not fully account for metabolic benefits, and that additional host-level factors may moderate probiotic efficacy.

While probiotics represent a targeted microbiota-modulating strategy with mechanistic evidence (immune modulation, barrier enhancement, and SCFA production), their clinical utility in MetS remains limited by modest benefits and inter-individual variability. Robust, long-term clinical trials are required to establish optimal strain selection, dosing strategies, and predictors of individual responsiveness.

**Table 2:** Summary of key studies examining probiotic interventions in metabolic syndrome (MetS).

Study	Model/Population	Formulation Details	Duration	Key Microbiota Changes	Metabolic/Clinical Outcomes
Wang et al. [124]	HFD-induced MetS mice	*Lactobacillus paracasei* CNCM I-4270; *Bifidobacterium animalis* subsp. *lactis* I-2494	12 weeks	↑ Bifidobacterium, Olsenella, Barnesiella, Allobaculum, Butyrivibrio, Lachnospiraceae; ↓ Alistipes, Desulfovibrionaceae, Oscillibacter, Clostridium, Dorea; attenuation of Proteobacteria expansion; ↑ acetate production	↓ body weight gain; ↓ blood glucose; improved glucose–insulin homeostasis; protection against hepatic steatosis; ↓ adipocyte size; ↓ TNF-α; ↓ endotoxemia (LBP)
Chen et al. [128]	HFD-induced MetS rats	*Bifidobacterium longum*	3 months	↑ Bifidobacterium abundance	↓ body weight, ↓ adiposity, ↓ glucose, ↓ TG, ↓ SBP; ↑ insulin sensitivity; ↓ LPS, ↓ IL-1β; improved gut barrier (↑ Reg I)
Chen et al. [129]	HFD-induced MetS rats	*Bifidobacterium adolescentis*	3 months	↑ Bifidobacterium abundance	↓ visceral fat, ↓ hepatic steatosis, ↑ insulin sensitivity
Tenorio-Jiménez et al. [130]	Humans (*n* = 25 MetS)	*Lactobacillus reuteri* V3401	12 weeks	↑ Verrucomicrobia; ↑ Akkermansia	↓ IL-6; ↓ sVCAM-1; no significant change in HOMA-IR
Xiao et al. [92]	Humans (*n* = 21 MetS)	*Bifidobacterium adolescentis* CCFM8630; *Lactobacillus reuteri* CCFM8631	11 weeks	↑ Erysipelotrichaceae, Fusobacteriaceae, Alcaligenaceae, Pasteurellaceae, Enterobacteriaceae; ↑ Fusobacterium, Phascolarctobacterium, Peptostreptococcus, Bacteroides, Lachnospira	Negative correlations: Phascolarctobacterium with glucose, insulin, hip circumference; Lachnospira and Bacteroides with fasting glucose
Bernini et al. [134]	Humans (*n* = 26 MetS)	Fermented milk with *Bifidobacterium lactis* HN019	45 days	Not reported	↓ BMI, ↓ total cholesterol, ↓ LDL-C, ↓ TNF-α, ↓ IL-6
Bernini et al. [135]	Humans (*n* = 19 MetS)	*B. lactis* HN019 supplementation	90 days	Not reported	↓ IL-6, ↓ homocysteine, ↓ hydroperoxides; ↑ adiponectin; ↑ NO metabolites
Numnark et al. [136]	Humans (*n* = 45 MetS)	*Bifidobacterium breve* BR03 + B632	3 months	Likely ↑ SCFA production (implied)	↓ BMI, ↓ waist circumference, ↓ visceral fat, ↓ glucose, ↓ HbA1c, ↓ TC/TG/LDL-C; ↑ HDL-C; no change in BP

↑ = increase and ↓ = decrease.

### 7.2. Prebiotics

Prebiotics are defined as non-digestible food components that selectively stimulate the growth and metabolic activity of beneficial gut bacteria, thereby conferring health benefits to the host [137]. These microbiota changes can also influence macronutrient absorption and metabolic processing by increasing fermentation of otherwise indigestible carbohydrates and enhancing pathways of carbohydrate metabolism, as demonstrated in human studies showing increased SCFA production and functional enrichment of carbohydrate metabolism genes after prebiotic intake [138]. Unlike probiotics, which introduce exogenous bacteria, prebiotics work indirectly by modifying the selective environment to favor endogenous beneficial taxa. This concept has since underpinned extensive research into dietary modulation of the gut microbiota. Prebiotics primarily consist of non-digestible carbohydrates, including polysaccharides such as inulin, resistant starches, cellulose, hemicelluloses, pectins, and gums, as well as certain oligosaccharides and unabsorbed sugars and sugar alcohols [139,140,141].

Among the most extensively studied prebiotics are fructooligosaccharides (FOS) and inulin, which are preferentially fermented by beneficial gut microorganisms, particularly *Bifidobacterium* spp. This selective fermentation promotes an increase in bifidobacterial abundance and activity, contributing to gut microbial balance and suppression of potentially pathogenic bacteria. Through these mechanisms, prebiotics support intestinal barrier function and overall gut health [139,140].

Prebiotics occur naturally in breast milk and are present in small quantities in various plant-based foods; however, they can also be produced as synthetic oligosaccharides [142,143,144]. Among synthetic prebiotics, fructooligosaccharides and galactooligosaccharides (GOS) are among the most widely investigated and have been shown to enhance the production of SCFAs while promoting the growth of beneficial genera such as *Bifidobacterium* and *Lactobacillus* [145]. Even short-term supplementation with prebiotics has been demonstrated to increase endogenous *Bifidobacteria*, which subsequently become predominant in human fecal microbiota. In addition to their effects on gut microbiota composition, prebiotics have been shown to modulate lipid metabolism, an effect that is largely attributed to the metabolic activity of fermentation-derived SCFAs [146].

Dietary sources rich in prebiotics include leeks, asparagus, chicory, Jerusalem artichokes, garlic, onions, wheat, oats, and soybeans [147]. Beyond gastrointestinal health, accumulating evidence suggests that prebiotics may exert protective metabolic effects. Delzenne et al. proposed that prebiotics can counteract several obesity-associated metabolic disturbances, including hyperglycemia, systemic inflammation, hepatic steatosis, and gut microbiota dysbiosis, particularly through increased abundance of *Bifidobacterium* spp. [148]. Notably, bifidobacterial levels have been inversely associated with fat mass accumulation, glucose intolerance, and circulating lipopolysaccharide (LPS) concentrations, implicating gut-derived endotoxemia in metabolic dysfunction [149].

Preclinical evidence demonstrates metabolic benefits of inulin supplementation in rodent models of high-fat diet-induced MetS [106]. In one study, inulin was administered orally at 10% in drinking water for 16 weeks, resulting in marked improvements in metabolic, inflammatory, and gut–liver axis dysfunction. Inulin supplementation significantly reduced body weight gain and improved glucose tolerance and insulin sensitivity. These effects were accompanied by reduced circulating triglycerides, low-density lipoprotein cholesterol, and free fatty acids, as well as decreased hepatic injury markers and systemic inflammatory cytokines, including IL 6, IL 1β, and TNF α [106].

At the tissue level, inulin attenuated hepatic steatosis and reduced white adipose tissue mass and adipocyte size. Mechanistically, these effects were associated with suppression of intestinal lipid absorption through downregulation of genes involved in lipid uptake and transport (Cd36, NPC1L1, and Apob48), alongside reduced hepatic lipogenesis due to decreased expression of ChREBP, Srebp 1c, FASN, and Scd 1. Inulin also modulated bile acid metabolism by reducing FGF15-mediated signaling, leading to increased fecal excretion of bile acids and triglycerides. At the microbial level, supplementation increased overall microbial diversity and shifted community composition toward a higher Bacteroidetes to Firmicutes ratio [106].

Consistent findings were observed in a high-carbohydrate, high-fat diet-induced MetS rat model further supplemented with fructose, in which a 5% inulin oligofructose-enriched diet was administered for eight weeks [150]. Supplementation reduced body weight gain, improved plasma lipid profiles, and decreased total abdominal fat mass. Marked improvements in gut morphology were also observed, including reduced ileal inflammation and improved villus and crypt architecture, alongside reduced hepatic lipid accumulation, inflammation, and liver enzyme levels. In addition, inulin improved glucose and insulin tolerance, lowered blood pressure, and ameliorated cardiovascular dysfunction, including impaired vascular reactivity and cardiac inflammation and fibrosis, indicating broad systemic benefits in this MetS model [150].

In a DS/obese rat model of MetS, inulin supplementation (5% or 20%) for four weeks primarily improved cardiovascular outcomes, reducing systolic blood pressure and ameliorating left ventricular diastolic dysfunction, cardiac inflammation, fibrosis, and macrophage infiltration, alongside reduced circulating IL 6 levels [151]. Inulin also attenuated hepatic steatosis, inflammation, and fibrosis, and partially improved hepatic metabolic regulation. In adipose tissue, inflammatory and fibrotic markers were reduced; however, effects on insulin signaling and adipocyte hypertrophy were limited. Notably, glucose tolerance and insulin resistance were unchanged, and plasma triglyceride levels were increased despite reductions in total and non-HDL cholesterol, indicating that metabolic benefits were modest and context-dependent in this model [151].

Clinical studies demonstrate more heterogeneous effects. In a six-month intervention in individuals with MetS, inulin supplementation significantly altered gut microbiota composition without affecting overall microbial diversity [152]. Specifically, an increased relative abundance of *Bacteroidetes*, particularly Bacteroides, was observed. Correlative analyses revealed positive associations between Bacteroidetes/Bacteroides abundance and HDL levels, and inverse associations between *Actinobacteria* and total and LDL cholesterol. However, no significant changes were observed in body weight, glycemic control, lipid profiles, or other clinical metabolic endpoints [152].

Another clinical study evaluating a 12-week multi-ingredient prebiotic fiber blend (10 g/day), including inulin, fructooligosaccharides, resistant dextrin, and guar gum derivatives, reported significant reductions in systemic inflammation, as evidenced by decreased hs CRP levels [153]. Participants also experienced improvements in perceived stress, anxiety, depression, and stress-related measures. Systolic blood pressure was significantly reduced, whereas lipid profiles and diastolic blood pressure remained unchanged. Microbiota analysis showed no major shifts in overall alpha or beta diversity; however, targeted analyses revealed enrichment of beneficial genera, including *Bifidobacterium* and *Parabacteroides*. Collectively, these findings suggest that prebiotic supplementation may confer anti-inflammatory and modest cardiometabolic benefits alongside selective modulation of gut microbiota composition, although effects on core MetS diagnostic parameters remain limited [153].

Overall, these findings support a role for prebiotics in mitigating metabolic dysregulation and suggest their potential utility in preventing or managing metabolic diseases. However, despite growing evidence linking prebiotic intake to improved metabolic outcomes, there remains a notable gap in the literature regarding their specific effects on gut microbiome composition and function in the context of MetS.

**Table 3:** Summary of key studies examining prebiotic interventions in metabolic syndrome (MetS).

Study	Model/Population	Formulation Details	Duration	Key Microbiota Changes	Metabolic/Clinical Outcomes
Huang et al. [106]	HFD-induced MetS mice	Inulin (10% *w*/*w* in drinking water)	16 weeks	↑ microbial diversity; ↑ Bacteroidetes/Firmicutes ratio	↓ body weight gain; ↑ glucose tolerance & insulin sensitivity; ↓ TG, LDL-C, free fatty acids; ↓ IL-6, IL-1β, TNF-α; ↓ hepatic steatosis; ↓ adiposity; ↓ intestinal lipid absorption; ↓ hepatic lipogenesis
Kumar et al. [150]	HCD-HFD + fructose-induced MetS rats	Inulin oligofructose (5% diet)	8 weeks	Not reported	↓ body weight; ↓ abdominal fat; improved lipid profile; ↓ liver fat & inflammation; ↑ insulin sensitivity; ↓ BP; improved cardiac function and vascular reactivity
Komatsu et al. [151]	DS/obese MetS rats	Inulin (5% or 20%)	4 weeks	Not reported	↓ systolic BP; improved cardiac inflammation, fibrosis & macrophage infiltration; ↓ liver steatosis & inflammation; ↓ adipose inflammation; no change in glucose tolerance/IR; ↑ triglycerides (adverse effect)
Tian et al. [152]	Humans (*n* = 20 MetS)	Inulin	6 months	↑ Bacteroidetes, especially Bacteroides	No significant changes in body weight, glucose, or lipid profile; microbiota–lipid correlations (Bacteroides ↑ HDL; Actinobacteria ↓ LDL/TC)
Hall et al. [153]	Humans (*n* = 40 MetS)	Multi-fiber blend (inulin + FOS + resistant dextrin + guar gum derivatives, 10 g/day)	12 weeks	↑ Bifidobacterium, Parabacteroides	↓ hs-CRP; ↓ stress, anxiety, depression; ↓ systolic BP; no change in lipids or diastolic BP

↑ = increase and ↓ = decrease.

### 7.3. Synbiotics

Synbiotics refer to formulations combining probiotics (live microorganisms) and prebiotics (selective growth substrates) that are designed to synergistically enhance the survival, colonization, and metabolic activity of beneficial microorganisms in the gastrointestinal tract [154]. By simultaneously providing live microorganisms and their selective substrates, synbiotics may exert additive or even synergistic effects on host health compared with prebiotics or probiotics administered alone [155]. Emerging evidence suggests that synbiotic interventions aimed at modulating the gut microbiota may support weight loss and improve metabolic outcomes [156,157,158]. Consequently, synbiotics have attracted increasing interest as potential dietary strategies for managing features of MetS. Nevertheless, despite encouraging clinical findings, there remains a paucity of studies that comprehensively characterize the effects of synbiotics on gut microbiome composition and function in individuals with MetS.

Clinical intervention studies provide evidence that synbiotic formulations improve multiple metabolic parameters in MetS, although microbiota compositional outcomes remain inconsistently reported. Eslamparast et al. conducted a 28-week randomized, double-blind, placebo-controlled study in 38 individuals with MetS (19 per group), evaluating a synbiotic formulation containing seven probiotic strains (*Lactobacillus casei*, *L. rhamnosus*, *Streptococcus thermophilus*, *Bifidobacterium breve*, *L. acidophilus*, *Bifidobacterium longum*, and *L. bulgaricus*), providing 2 × 10^8^ CFU per capsule, combined with fructo-oligosaccharides [159]. Participants received the synbiotic twice daily. This intervention resulted in significant improvements in fasting blood glucose and insulin resistance, alongside reductions in triglyceride and total cholesterol concentrations and an increase in HDL cholesterol. However, no significant changes were observed in LDL cholesterol, waist circumference, body mass index, metabolic equivalent of task, or energy intake, indicating that metabolic benefits occurred independently of measurable changes in adiposity or physical activity [159].

Similarly, Rabiei et al. conducted a 12-week triple-blinded randomized clinical trial in 46 individuals with MetS. Participants received either a synbiotic capsule or a placebo capsule twice daily. Each synbiotic capsule contained seven probiotic strains, providing a total viable count of 2 × 10^8^ CFU per capsule, combined with fructooligosaccharides. The intervention resulted in significant improvements in BMI, fasting blood glucose, insulin resistance, and homeostatic model assessment of insulin resistance (HOMA-IR). In contrast, inflammatory biomarkers such as high-sensitivity C-reactive protein (hs-CRP) and IL-6 were not significantly altered, suggesting that the observed metabolic improvements may not be mediated through measurable changes in systemic inflammation over the intervention period [160].

Further evidence is provided by Cicero et al., who reported that daily (once-daily) consumption of a synbiotic formulation containing *Lactobacillus plantarum* PBS067, *Lactobacillus acidophilus* PBS066, and *Lactobacillus reuteri* PBS072, each at 2 × 10^9^ CFU per strain per dose (total 6 × 10^9^ CFU per dose), combined with active prebiotics, for two months in 60 elderly individuals with MetS resulted in significant reductions in waist circumference, fasting plasma insulin, total cholesterol, triglycerides, mean arterial pressure, LDL cholesterol, hs-CRP, and tumor necrosis factor-α (TNF-α) serum levels [161]. However, no significant changes were observed in HOMA-IR, hepatic steatosis index, or leptin-to-adiponectin ratio, highlighting variability in metabolic responsiveness and underscoring the complexity of synbiotic effects across different cardiometabolic endpoints [161].

Across these three synbiotic trials, metabolic improvements in glycemic control, lipid metabolism, and inflammatory markers are relatively consistent. However, none of these studies reported detailed microbiota compositional changes following intervention. This represents a significant gap in understanding whether metabolic benefits are mediated through predicted shifts in microbial composition or through alternative mechanisms. Heterogeneity in strain formulations, CFU dosages, and intervention durations (12–28 weeks) further complicates cross-study synthesis and limits the ability to identify which synbiotic components drive metabolic benefits. While synbiotics show promise for improving MetS-related metabolic parameters, the absence of mechanistic microbiota profiling hampers interpretation. Robust future studies incorporating high-resolution microbiome and metabolomic analyses are required to determine whether microbiota-mediated mechanisms underpin clinical benefits, and to identify optimal strain composition and dosing strategies.

### 7.4. Postbiotics

Postbiotics are defined as non-viable microbial cells and/or their components, including intact non-viable microorganisms, microbial cell fragments, and associated metabolites present in a finished product [162]. These non-viable microbes are produced through controlled, reproducible inactivation processes such as heat treatment, radiation, high pressure, or cell lysis [162,163]. Common examples include heat-killed and lyophilized *Lactobacillus acidophilus* strain LB [164], heat-killed *Bifidobacterium bifidum* MIMBb75 [165], pasteurized *Akkermansia muciniphila* [121], and fermented culture media.

Postbiotics exhibit immunomodulatory, anti-inflammatory, and antioxidant activities [166,167]. They have been shown to strengthen intestinal barrier integrity and function [168], enhance nutrient absorption by promoting digestive enzymes [169], and modulate microbiota composition by selectively promoting beneficial bacteria while inhibiting pathogenic species [170], which is a process critical for maintaining gut health. In individuals with metabolic disorders, postbiotics have been associated with reduced serum LDL cholesterol, lower BMI, improved insulin resistance (HOMA-IR), and decreased hepatic fat content [121,171].

In a MetS rat model, animals were treated orally with heat-killed *Lactobacillus plantarum* L-137 (HK L-137) at low (2 mg/kg) or high (75 mg/kg) doses for 4 weeks [172]. Body weight, food and water intake, systolic blood pressure, organ mass (heart, liver, kidney, visceral fat, and BAT), and serum lipid levels (total cholesterol, LDL-C, and triglycerides) were not significantly improved by treatment. However, the low dose (2 mg/kg) showed selective protective effects on diastolic cardiac function, including reduced LV filling abnormalities and improved relaxation indices.

At the tissue level, HK L-137 exerted anti-inflammatory and anti-fibrotic effects, particularly in cardiovascular and adipose tissues [172]. It reduced macrophage infiltration, collagen deposition, oxidative stress, and inflammatory gene expression (e.g., TNF-α, MCP-1, IL-1β, and IL-12) in the heart and adipose depots. These changes were accompanied by modulation of signaling pathways such as NF-κB and ERK1/2, suggesting attenuation of inflammatory signaling despite unchanged systemic metabolic outcomes [172]. Importantly, HK L-137 improved whole-body insulin sensitivity, as shown by insulin tolerance testing, and reduced fasting insulin and HOMA-β in MetS rats, with stronger effects generally observed at the low dose [172]. In the liver, it suppressed inflammatory markers and genes involved in gluconeogenesis and lipid synthesis, indicating improved hepatic metabolic regulation, even though circulating adiponectin and AMPK/Akt signaling were largely unchanged [172]. Overall, in the MetS group, HK L-137 mainly reduced inflammation and improved insulin sensitivity with some organ-specific benefits (heart, adipose tissue, and liver), but it did not significantly improve body weight, lipid levels, or blood pressure.

In a human clinical trial, individuals with MetS receiving oral sodium butyrate (4 g/day for 4 weeks) showed limited metabolic improvement [173]. Butyrate treatment paradoxically increased plasma total and LDL cholesterol, and did not improve insulin sensitivity, glucose homeostasis, or energy metabolism despite severe baseline insulin resistance. Fecal SCFA concentrations (acetate, propionate, and butyrate) significantly decreased, whilst brown adipose tissue activity and bile acid profiles remained unchanged [173]. Microbiome analysis revealed no major shifts in overall diversity or community structure; however, specific taxa (Coriobacteriaceae and Clostridiales) were altered and correlated with hepatic insulin resistance and plasma LDL cholesterol. Overall, oral butyrate supplementation did not improve insulin sensitivity or energy metabolism and was associated with an adverse lipid response, demonstrating minimal systemic metabolic benefit despite local microbiome changes.

Postbiotics demonstrate promising tissue-protective effects in MetS, primarily through reducing inflammation and oxidative stress whilst enhancing gut barrier integrity and insulin signaling. However, these local benefits do not consistently translate to systemic metabolic improvements, as effects on body weight, lipid profiles, glucose control, and blood pressure remain mixed, ranging from clear benefits in animal models to limited or negligible effects in humans. Critically, the literature lacks robust evidence on postbiotics’ specific effects on gut microbiome composition and function in the context of MetS.

### 7.5. Fecal Microbiota Transplantation (FMT)

FMT is a therapeutic approach that involves the transfer of processed fecal material from a healthy donor into the gastrointestinal tract of a recipient with the aim of reconstituting microbial communities from a dysbiotic state toward a healthy, functionally diverse ecosystem [174]. Unlike probiotics (introducing single or multiple selected strains) or prebiotics (modifying selective nutrient availability), FMT represents a comprehensive microbiota reconstitution strategy, introducing hundreds to thousands of bacterial taxa in a single intervention [175]. By reconstituting microbial communities, FMT has been explored as a treatment for a range of gastrointestinal and systemic diseases associated with gut microbiota dysbiosis, including MetS [115].

Early mechanistic studies demonstrate that FMT can induce rapid shifts in microbial composition and improve insulin sensitivity through SCFA- and amino acid-mediated pathways. Early evidence supporting the metabolic effects of FMT was provided by Vrieze et al., who investigated the impact of FMT from lean donors into male recipients with MetS [176]. Six weeks after transplantation, recipients exhibited a significant improvement in peripheral insulin sensitivity, accompanied by increased gut microbial diversity. This metabolic improvement was associated with a higher abundance of butyrate-producing bacterial taxa, including *Roseburia intestinalis* and *Eubacterium hallii*. Importantly, these changes occurred without alterations in total fecal bacterial or archaeal counts. Moreover, no significant differences were observed in dietary intake, resting energy expenditure, or counter-regulatory hormone levels, suggesting that the observed metabolic benefits were mediated primarily through microbiota-dependent mechanisms rather than changes in lifestyle or energy balance [176].

These findings were extended by Kootte et al., who demonstrated that FMT from lean donors to individuals with obesity-associated MetS altered gut microbiota composition and improved insulin sensitivity [177]. The authors proposed that changes in circulating plasma metabolites—particularly amino acids such as γ-aminobutyric acid (GABA)—resulting from microbiota restructuring could underlie the metabolic benefits of FMT [177]. Of particular interest was an increase in *Lactobacillus brevis*, a bacterial species known to produce GABA [178]. Experimental evidence supports the metabolic relevance of this pathway, as incorporation of *L. brevis* into insulin-resistant rat models improved glucose homeostasis [179], and exogenous GABA supplementation has been shown to enhance insulin sensitivity in rodents [180].

In addition to GABA-related pathways, improved insulin sensitivity following FMT was associated with increased abundance of SCFA-producing bacteria, particularly members of the genus *Eubacterium* [176,181]. SCFAs are known to stimulate the secretion of glucagon-like peptide-1 (GLP-1) and peptide YY (PYY), gut-derived hormones that play key roles in glucose homeostasis by enhancing insulin secretion, improving insulin sensitivity, and regulating appetite by acting as satiety factors [182,183,184]. Despite these functional improvements, neither study reported significant changes in body weight, caloric intake, resting energy expenditure, or routine plasma biochemical parameters following FMT. Furthermore, Kootte et al. observed no significant changes in overall fecal microbial diversity, highlighting that specific functional shifts within the microbiota, rather than broad diversity changes, may be sufficient to elicit metabolic benefits [177].

FMT represents a broad-spectrum microbiota-modulating intervention capable of inducing rapid and functionally meaningful shifts in gut microbial composition. However, its metabolic benefits in MetS appear variable and may be transient. Therapeutic outcomes are highly donor-dependent, which complicates standardization and reproducibility. In addition, FMT carries potential risks, including transmission of pathogens, transfer of antimicrobial resistance genes, and uncertain long-term effects on the recipient’s microbial ecosystem. Regulatory constraints and mechanistic complexity further limit its widespread clinical application. Although FMT shows promise, particularly in improving insulin sensitivity—potentially through microbiota-mediated modulation of SCFA production, amino acid metabolism, and gut hormone secretion—robust long-term clinical studies are still required to confirm its safety, durability, and feasibility in MetS.

**Table 4:** Summary of key studies examining synbiotic, postbiotic, and fecal microbiota transplantation (FMT) interventions in metabolic syndrome (MetS).

Intervention Type	Study	Model/Population	Formulation Details	Duration	Key Microbiota Changes	Metabolic/Clinical Outcomes
Synbiotics	Eslamparast et al. [159]	Humans (*n* = 38, MetS)	7 probiotic strains; 2 × 10^8^ CFU/capsule + FOS	28 weeks	Not reported	↓ fasting glucose, ↓ insulin resistance, ↓ TG, ↓ total cholesterol; ↑ HDL; no change in LDL, waist circumference, BMI
Synbiotics	Rabiei et al. [160]	Humans (*n* = 46, MetS)	7 probiotic strains; 2 × 10^8^ CFU/capsule + FOS	12 weeks	Not reported	↓ BMI, ↓ fasting glucose, ↓ insulin resistance, ↓ HOMA-IR; no change in hs-CRP or IL-6
Synbiotics	Cicero et al. [161]	Elderly humans (*n* = 60, MetS)	3 probiotic strains; 2 × 10^9^ CFU/strain + prebiotics	2 months	Not reported	↓ waist circumference, ↓ insulin, ↓ TC, ↓ TG, ↓ MAP, ↓ LDL-C, ↓ hs-CRP, ↓ TNF-α; no change in HOMA-IR, hepatic steatosis index, leptin/adiponectin ratio
Postbiotics	Uchinaka et al. [172]	MetS rat model	Heat-killed *Lactobacillus plantarum* L-137 (2 or 75 mg/kg)	4 weeks	Not reported	No change in body weight, lipids, or BP; improved diastolic cardiac function (low dose); ↓ inflammation, fibrosis, oxidative stress; ↑ insulin sensitivity; improved hepatic metabolism (dose-dependent)
Postbiotics	Bouter et al. [173]	Humans (*n* = 10 MetS)	Sodium butyrate (4 g/day)	4 weeks	↓ fecal SCFAs; altered Coriobacteriaceae & Clostridiales	↑ total cholesterol and LDL-C; no improvement in insulin sensitivity, energy metabolism, BAT activity, or bile acids
FMT	Vrieze et al. [176]	Humans (male MetS)	Lean donor fecal microbiota	6 weeks	↑ *Roseburia intestinalis*, *Eubacterium hallii*; ↑ diversity	↑ insulin sensitivity; no change in weight or energy expenditure
FMT	Kootte et al. [177]	Humans (obesity + MetS)	Lean donor fecal microbiota	18 weeks	↑ *Lactobacillus brevis*; ↑ *Eubacterium* spp.; altered metabolome	↑ insulin sensitivity; no change in weight or overall diversity

↑ = increase and ↓ = decrease.

## 8. Conclusions and Future Perspectives

Over the past decade, the gut microbiota has emerged as a central regulator of metabolic health, shaping host physiology through its influence on nutrient metabolism, immune function, and inflammatory signaling. As highlighted throughout this review, disturbances in gut microbial composition—driven by modern dietary patterns, lifestyle factors, and environmental exposures—are increasingly recognized as integral to the development and progression of MetS. Rather than acting in isolation, these microbial alterations intersect with core features of MetS, including insulin resistance, dyslipidemia, low-grade chronic inflammation, and impaired gut barrier integrity.

A recurring theme across human and animal studies is the convergence toward a dysbiotic gut microbial profile in MetS, characterized by reduced microbial diversity, depletion of beneficial SCFA-producing bacteria, and an enrichment of pro-inflammatory and endotoxin-producing taxa. These changes promote metabolic endotoxemia and inflammatory cascades that exacerbate cardiometabolic risk. Viewed from this perspective, the gut microbiota represents not merely a biomarker of metabolic dysfunction, but an active participant in disease pathogenesis and, importantly, a modifiable therapeutic target. Specifically, dysbiosis-targeted interventions could potentially address these common dysbiotic features, even in patients with phenotypically diverse MetS, provided interventions are tailored to achieve the specific compositional and functional goals (restored SCFA production, reduced LPS translocation, and rebalanced immune responses) identified as dysbiotic hallmarks.

Despite growing interest in microbiota-targeted interventions, important limitations in the current evidence base remain. Although a substantial number of studies have examined probiotics, prebiotics, synbiotics, postbiotics, and FMT in the context of obesity and MetS, relatively few have comprehensively characterized accompanying changes in gut microbiota composition and function. Many investigations primarily report metabolic or clinical outcomes, limiting mechanistic insight into how microbiota modulation contributes to observed effects. Moreover, clinical responses to these interventions are highly heterogeneous, reflecting the complexity of host–microbe interactions and the influence of factors such as baseline microbiota composition, dietary intake, host genetics, and disease severity. The evidence base is further constrained by a relative paucity of well-powered studies conducted specifically in adults with established MetS, which restricts definitive conclusions regarding clinical efficacy. Collectively, these limitations suggest that microbiota-based interventions are unlikely to be universally effective as standalone therapies but may have greater therapeutic relevance when integrated with established lifestyle and pharmacological approaches.

Advancing the clinical translation of microbiome research in MetS requires a shift away from one-size-fits-all approaches toward precision-based therapeutic strategies. In this approach, interventions are tailored to individual patient characteristics, with stratification based on baseline gut microbial composition, functional capacity, and metabolic phenotype to identify distinct responder subgroups. Emerging multi-omics technologies, including metagenomics, metabolomics, and transcriptomics, provide the necessary framework to define predictive microbial signatures and functionally relevant metabolic pathways that can guide therapy selection.

Precision microbiome interventions may include strain-specific probiotics targeting defined deficiencies in SCFA-producing bacteria, tailored prebiotics designed to selectively enrich beneficial taxa, synbiotics delivering targeted microbial substrates and metabolites, and optimized donor selection strategies in FMT. Importantly, the goal of these approaches extends beyond compositional changes to restoration of key microbial functions, including SCFA production, reduction of metabolic endotoxemia, enhancement of gut barrier integrity, and modulation of immune signaling.

This precision-focused strategy is expected to reduce inter-individual variability in treatment response, improve therapeutic efficacy, and support the development of personalized microbiome-based therapies for MetS.

In this evolving landscape, emerging approaches, including postbiotics and next-generation probiotics with defined functional properties, offer promising avenues for overcoming current limitations. However, the full potential of precision microbiota strategies will be realized only through mechanistic integration by combining multi-omics stratification with a deep understanding of dysbiosis-specific mechanisms that will enable microbiome science to move from broad dysbiosis reversal toward truly personalized, mechanism-matched interventions for MetS prevention and management.

## Data Availability

No new data were generated or analyzed in this study.

## References

[1] Lambert A.J., Merry B.J. (2004). Effect of caloric restriction on mitochondrial reactive oxygen species production and bioenergetics: Reversal by insulin. Am. J. Physiol.-Regul. Integr. Comp. Physiol..

[2] Oda E. (2018). Historical perspectives of the metabolic syndrome. Clin. Dermatol..

[3] Qin Q., Yan S., Yang Y., Chen J., Li T., Gao X., Yan H., Wang Y., Wang J., Wang S. (2021). A Metagenome-Wide Association Study of the Gut Microbiome and Metabolic Syndrome. Front. Microbiol..

[4] Duncan S.H., Lobley G.E., Holtrop G., Ince J., Johnstone A.M., Louis P., Flint H.J. (2008). Human colonic microbiota associated with diet, obesity and weight loss. Int. J. Obes..

[5] Jandhyala S.M., Talukdar R., Subramanyam C., Vuyyuru H., Sasikala M., Reddy D.N. (2015). Role of the normal gut microbiota. World J. Gastroenterol. WJG.

[6] Lam Y.Y., Ha C.W.Y., Campbell C.R., Mitchell A.J., Dinudom A., Oscarsson J., Cook D.I., Hunt N.H., Caterson I.D., Holmes A.J. (2012). Increased gut permeability and microbiota change associate with mesenteric fat inflammation and metabolic dysfunction in diet-induced obese mice. PLoS ONE.

[7] Mishra S.P., Wang B., Jain S., Ding J., Rejeski J., Furdui C.M., Kitzman D.W., Taraphder S., Brechot C., Kumar A. (2023). A mechanism by which gut microbiota elevates permeability and inflammation in obese/diabetic mice and human gut. Gut.

[8] Crudo F., Aichinger G., Mihajlovic J., Varga E., Dellafiora L., Warth B., Dall’Asta C., Berry D., Marko D. (2021). In vitro interactions of Alternaria mycotoxins, an emerging class of food contaminants, with the gut microbiota: A bidirectional relationship. Arch. Toxicol..

[9] Hildebrandt M.A., Hoffmann C., Sherrill–Mix S.A., Keilbaugh S.A., Hamady M., Chen Y.Y., Knight R., Ahima R.S., Bushman F., Wu G.D. (2009). High-fat diet determines the composition of the murine gut microbiome independently of obesity. Gastroenterology.

[10] Liu Q., Shao W., Zhang C., Xu C., Wang Q., Liu H., Sun H., Jiang Z., Gu A. (2017). Organochloride pesticides modulated gut microbiota and influenced bile acid metabolism in mice. Environ. Pollut..

[11] Mikkelsen K.H., Frost M., Bahl M.I., Licht T.R., Jensen U.S., Rosenberg J., Pedersen O., Hansen T., Rehfeld J.F., Holst J.J. (2015). Effect of antibiotics on gut microbiota, gut hormones and glucose metabolism. PLoS ONE.

[12] Pinget G., Tan J., Janac B., Kaakoush N.O., Angelatos A.S., O’Sullivan J., Koay Y.C., Sierro F., Davis J., Divakarla S.K. (2019). Impact of the food additive titanium dioxide (E171) on gut microbiota-host interaction. Front. Nutr..

[13] Tian Y., Gui W., Rimal B., Koo I., Smith P.B., Nichols R.G., Cai J., Liu Q., Patterson A.D. (2020). Metabolic impact of persistent organic pollutants on gut microbiota. Gut Microbes.

[14] Yan W., Hamid N., Deng S., Jia P.-P., Pei D.-S. (2020). Individual and combined toxicogenetic effects of microplastics and heavy metals (Cd, Pb, and Zn) perturb gut microbiota homeostasis and gonadal development in marine medaka (*Oryzias melastigma*). J. Hazard. Mater..

[15] Miao Z.H., Zhou W.X., Cheng R.Y., Liang H.J., Jiang F.L., Shen X., Lu J.H., Li M., He F. (2021). Dysbiosis of intestinal microbiota in early life aggravates high-fat diet induced dysmetabolism in adult mice. BMC Microbiol..

[16] Ding Z., Ren K., Xu Y., Feng T., Cui K., Liu Q., Liao C. (2026). Disease-driven restructuring of the gut microbiome underlies inflammatory bowel disease dysbiosis. Front. Microbiol..

[17] Lee J., Oh S.J., Ha E., Shin G.Y., Kim H.J., Kim K., Lee C.K. (2024). Gut microbial and human genetic signatures of inflammatory bowel disease increase risk of comorbid mental disorders. npj Genom. Med..

[18] Munoz-Pinto M.F., Candeias E., Melo-Marques I., Esteves A.R., Maranha A., Magalhães J.D., Carneiro D.R., Sant’Anna M., Pereira-Santos A.R., Abreu A.E. (2024). Gut-first Parkinson’s disease is encoded by gut dysbiome. Mol. Neurodegener..

[19] Zhao H., Zhou X., Song Y., Zhao W., Sun Z., Zhu J., Yu Y. (2025). Multi-omics analyses identify gut microbiota-fecal metabolites-brain-cognition pathways in the Alzheimer’s disease continuum. Alzheimer’s Res. Ther..

[20] Ruiz-Limón P., Mena-Vázquez N., Moreno-Indias I., Lisbona-Montañez J.M., Mucientes A., Manrique-Arija S., Redondo-Rodriguez R., Cano-García L., Tinahones F.J., Fernández-Nebro A. (2025). Gut dysbiosis is associated with difficult-to-treat rheumatoid arthritis. Front. Med..

[21] Sun G., Zhao S., Huang H., Guan W., Wang X., Zhang H., Zhang M., Hou D., Xu C., Chai R. (2025). Integrated gut microbiome and metabolomics analysis reveals microbial-metabolic cross-talk in allergic rhinitis. Front. Microbiol..

[22] Morsy Y., Shafie N.S., Amer M. (2025). Integrative analysis of gut microbiota and metabolic pathways reveals key microbial and metabolomic alterations in diabetes. Sci. Rep..

[23] Wang X., Guo Q., Liu Z., Wang Y., Cao C., Jin L., Li C., Xiao J., Zhao W. (2024). Alterations in the gut microbiota composition in obesity with and without type 2 diabetes: A pilot study. Diabetes Metab. Syndr. Obes..

[24] Ling Z., Ding W., Liu X., Zhang J., Cheng Y., Zhu Z., Wu L., Xu X., Gao Y., Jiang R. (2025). Gut microbiota dysbiosis and systemic immune dysfunction in critical ill patients with multidrug-resistant bacterial colonization and infection. J. Transl. Med..

[25] Remely M., Aumueller E., Jahn D., Hippe B., Brath H., Haslberger A.G. (2014). Microbiota and epigenetic regulation of inflammatory mediators in type 2 diabetes and obesity. Benef. Microbes.

[26] Magne F., Gotteland M., Gauthier L., Zazueta A., Pesoa S., Navarrete P., Balamurugan R. (2020). The firmicutes/bacteroidetes ratio: A relevant marker of gut dysbiosis in obese patients?. Nutrients.

[27] Chanda D., De D. (2024). Meta-analysis reveals obesity associated gut microbial alteration patterns and reproducible contributors of functional shift. Gut Microbes.

[28] Nowak E.C., de Vries V.C., Wasiuk A., Ahonen C., Bennett K.A., Le Mercier I., Ha D.-G., Noelle R.J. (2012). Tryptophan hydroxylase-1 regulates immune tolerance and inflammation. J. Exp. Med..

[29] Chansa O., Shantavasinkul P.C., Monsuwan W., Sirivarasai J. (2024). Association between gut microbiota profiles, dietary intake, and inflammatory markers in overweight and obese women. Foods.

[30] Ling Z., Lan Z., Cheng Y., Liu X., Li Z., Yu Y., Wang Y., Shao L., Zhu Z., Gao J. (2024). Altered gut microbiota and systemic immunity in Chinese patients with schizophrenia comorbid with metabolic syndrome. J. Transl. Med..

[31] Zhang X., Shi L., Sun T., Guo K., Geng S. (2021). Dysbiosis of gut microbiota and its correlation with dysregulation of cytokines in psoriasis patients. BMC Microbiol..

[32] Belizário J.E., Faintuch J., Garay-Malpartida M. (2018). Gut microbiome dysbiosis and immunometabolism: New frontiers for treatment of metabolic diseases. Mediat. Inflamm..

[33] López-Montoya P., Rivera-Paredez B., Palacios-González B., Morán-Ramos S., López-Contreras B.E., Canizales-Quinteros S., Salmerón J., Velázquez-Cruz R. (2023). Dietary Patterns Are Associated with the Gut Microbiome and Metabolic Syndrome in Mexican Postmenopausal Women. Nutrients.

[34] Amador-Lara F., Andrade-Villanueva J.F., Vega-Magaña N., Peña-Rodríguez M., Alvarez-Zavala M., Sanchez-Reyes K., Toscano-Piña M., Peregrina-Lucano A.A., del Toro-Arreola S., González-Hernández L.A. (2022). Gut microbiota from Mexican patients with metabolic syndrome and HIV infection: An inflammatory profile. J. Appl. Microbiol..

[35] Newman N.K., Zhang Y., Padiadpu J., Miranda C.L., Magana A.A., Wong C.P., Hioki K.A., Pederson J.W., Li Z., Gurung M. (2023). Reducing gut microbiome-driven adipose tissue inflammation alleviates metabolic syndrome. Microbiome.

[36] Sun P., Wang M., Liu Y.-X., Li L., Chai X., Zheng W., Chen S., Zhu X., Zhao S. (2023). High-fat diet-disturbed gut microbiota-colonocyte interactions contribute to dysregulating peripheral tryptophan-kynurenine metabolism. Microbiome.

[37] Rosendo-Silva D., Viana S., Carvalho E., Reis F., Matafome P. (2023). Are gut dysbiosis, barrier disruption, and endotoxemia related to adipose tissue dysfunction in metabolic disorders? Overview of the mechanisms involved. Intern. Emerg. Med..

[38] Guevara-Cruz M., Flores-Lopez A.G., Aguilar-Lopez M., Sanchez-Tapia M., Medina-Vera I., Dıaz D., Tovar A.R., Torres N. (2019). Improvement of lipoprotein profile and metabolic endotoxemia by a lifestyle intervention that modifies the gut microbiota in subjects with metabolic syndrome. J. Am. Heart Assoc..

[39] Zhong X., Harrington J.M., Millar S.R., Perry I.J., O’Toole P.W., Phillips C.M. (2020). Gut microbiota associations with metabolic health and obesity status in older adults. Nutrients.

[40] Wutthi-In M., Cheevadhanarak S., Yasom S., Kerdphoo S., Thiennimitr P., Phrommintikul A., Chattipakorn N., Kittichotirat W., Chattipakorn S. (2020). Gut microbiota profiles of treated metabolic syndrome patients and their relationship with metabolic health. Sci. Rep..

[41] Zhou Q., Pang G., Zhang Z., Yuan H., Chen C., Zhang N., Yang Z., Sun L. (2021). Association between gut Akkermansia and metabolic syndrome is dose-dependent and affected by microbial interactions: A cross-sectional study. Diabetes Metab. Syndr. Obes..

[42] Gong Y., Jin X., Yuan B., Lv Y., Yan G., Liu M., Xie C., Liu J., Tang Y., Gao H. (2021). G protein-coupled receptor 109A maintains the intestinal integrity and protects against ETEC mucosal infection by promoting IgA secretion. Front. Immunol..

[43] Peng K., Xiao S., Xia S., Li C., Yu H., Yu Q. (2024). Butyrate inhibits the HDAC8/NF-κB pathway to enhance Slc26a3 expression and improve the intestinal epithelial barrier to relieve colitis. J. Agric. Food Chem..

[44] Zhang Y., Lei Y., Honarpisheh M., Kemter E., Wolf E., Seissler J. (2021). Butyrate and class I histone deacetylase inhibitors promote differentiation of neonatal porcine islet cells into beta cells. Cells.

[45] Haumaitre C., Lenoir O., Scharfmann R. (2008). Histone deacetylase inhibitors modify pancreatic cell fate determination and amplify endocrine progenitors. Mol. Cell. Biol..

[46] Li H., Gao Z., Zhang J., Ye X., Xu A., Ye J., Jia W. (2012). Sodium butyrate stimulates expression of fibroblast growth factor 21 in liver by inhibition of histone deacetylase 3. Diabetes.

[47] Jung T.-H., Han K.-S., Park J.-H., Hwang H.-J. (2022). Butyrate modulates mucin secretion and bacterial adherence in LoVo cells via MAPK signaling. PLoS ONE.

[48] Meissner S., Hagen F., Deiner C., Günzel D., Greco G., Shen Z., Aschenbach J.R. (2017). Key role of short-chain fatty acids in epithelial barrier failure during ruminal acidosis. J. Dairy Sci..

[49] Yue X., Wen S., Long-Kun D., Man Y., Chang S., Min Z., Shuang-Yu L., Xin Q., Jie M., Liang W. (2022). Three important short-chain fatty acids (SCFAs) attenuate the inflammatory response induced by 5-FU and maintain the integrity of intestinal mucosal tight junction. BMC Immunol..

[50] Zhao Y., Chen J., Qin Y., Yuan J., Yu Z., Ma R., Liu F., Zhao J. (2025). Linking Short-Chain Fatty Acids to Systemic Homeostasis: Mechanisms, Therapeutic Potential, and Future Directions. J. Nutr. Metab..

[51] Lei L., Deng D., Xu W., Yue M., Wu D., Fu K., Shi Z. (2024). Increased intestinal permeability and lipopolysaccharide contribute to swainsonine-induced systemic inflammation. Ecotoxicol. Environ. Saf..

[52] Zhou X., Li J., Guo J., Geng B., Ji W., Zhao Q., Li J., Liu X., Liu J., Guo Z. (2018). Gut-dependent microbial translocation induces inflammation and cardiovascular events after ST-elevation myocardial infarction. Microbiome.

[53] Wei X., Yi X., Liu J., Sui X., Li L., Li M., Lv H., Yi H. (2024). Circ-phkb promotes cell apoptosis and inflammation in LPS-induced alveolar macrophages via the TLR4/MyD88/NF-kB/CCL2 axis. Respir. Res..

[54] Zamora R., Chavan S., Zanos T., Simmons R.L., Billiar T.R., Vodovotz Y. (2021). Spatiotemporally specific roles of TLR4, TNF, and IL-17A in murine endotoxin-induced inflammation inferred from analysis of dynamic networks. Mol. Med..

[55] Zhang C., Teng X., Cao Q., Deng Y., Yang M., Wang L., Rui D., Ling X., Wei C., Chen Y. (2025). Gut microbiota dysbiosis exacerbates heart failure by the LPS-TLR4/NF-κB signalling axis: Mechanistic insights and therapeutic potential of TLR4 inhibition. J. Transl. Med..

[56] Kim J.J., Sears D.D. (2010). TLR4 and insulin resistance. Gastroenterol. Res. Pract..

[57] Liang H., Sathavarodom N., Colmenares C., Gelfond J., Espinoza S.E., Ganapathy V., Musi N. (2022). Effect of acute TLR4 inhibition on insulin resistance in humans. J. Clin. Investig..

[58] Tian X., Zhang P. (2025). TLR4 modulates simvastatin’s impact on HDL cholesterol and glycemic control. Front. Pharmacol..

[59] Ishchenko A., Van Mechelen M., Pazmino S., Storms L., Neerinckx B., Verschueren P., Lories R., de Vlam K. (2025). Low apolipoprotein A1 and high apolipoprotein B levels indicate specific lipid changes in treatment naïve early psoriatic arthritis. RMD Open.

[60] Wang C., Zheng M., Yun C., Feng Z., Li Y., Chen S., Wu S., Xue H. (2025). Joint and Temporal Relationships of Systemic Inflammation and Atherogenic Dyslipidemia with Risk of Cardiometabolic Disease: A Longitudinal Cohort Study. J. Inflamm. Res..

[61] Li J., Wei W., Lin S.I., Tan W., Lai Z., Tu Y.U., Li Y. (2024). Bufrudin Reduces Atherosclerosis in Apolipoprotein E Knockout Mice by Modulating the Toll-Like Receptor 4/Nuclear Factor Kappa B/p38 Mitogen-Activated Protein Kinase Signalling Pathway in the Aorta. Indian J. Pharm. Sci..

[62] Lu Z., Zhang X., Li Y., Jin J., Huang Y. (2013). TLR4 antagonist reduces early-stage atherosclerosis in diabetic apolipoprotein E-deficient mice. J. Endocrinol..

[63] Peña-Durán E., García-Galindo J.J., López-Murillo L.D., Huerta-Huerta A., Balleza-Alejandri L.R., Beltrán-Ramírez A., Anaya-Ambriz E.J., Suárez-Rico D.O. (2025). Microbiota and inflammatory markers: A review of their interplay, clinical implications, and metabolic disorders. Int. J. Mol. Sci..

[64] Cortés-Martín A., Iglesias-Aguirre C.E., Meoro A., Selma M.V., Espín J.C. (2020). There is no distinctive gut microbiota signature in the metabolic syndrome: Contribution of cardiovascular disease risk factors and associated medication. Microorganisms.

[65] Chang Y., Chen Y., Zhou Q., Wang C., Chen L., Di W., Zhang Y. (2020). Short-chain fatty acids accompanying changes in the gut microbiome contribute to the development of hypertension in patients with preeclampsia. Clin. Sci..

[66] Jia Q., Yu X., Chang Y., You Y., Chen Z., Wang Y., Liu B., Chen L., Ma D., Xing Y. (2022). Dynamic changes of the gut microbiota in preterm infants with different gestational age. Front. Microbiol..

[67] Wang J., Gu X., Yang J., Wei Y., Zhao Y. (2019). Gut microbiota dysbiosis and increased plasma LPS and TMAO levels in patients with preeclampsia. Front. Cell. Infect. Microbiol..

[68] Hall A.B., Yassour M., Sauk J., Garner A., Jiang X., Arthur T., Lagoudas G.K., Vatanen T., Fornelos N., Wilson R. (2017). A novel *Ruminococcus gnavus* clade enriched in inflammatory bowel disease patients. Genome Med..

[69] Qu Q., Dou Q., Xiang Z., Yu B., Chen L., Fan Z., Zhao X., Yang S., Zeng P. (2025). Population-level gut microbiome and its associations with environmental factors and metabolic disorders in Southwest China. npj Biofilms Microbiomes.

[70] Chen Z., Radjabzadeh D., Chen L., Kurilshikov A., Kavousi M., Ahmadizar F., Ikram M.A., Uitterlinden A.G., Zhernakova A., Fu J. (2021). Association of insulin resistance and type 2 diabetes with gut microbial diversity: A microbiome-wide analysis from population studies. JAMA Netw. Open.

[71] Pedersen H.K., Gudmundsdottir V., Nielsen H.B., Hyotylainen T., Nielsen T., Jensen B.A., Forslund K., Hildebrand F., Prifti E., Falony G. (2016). Human gut microbes impact host serum metabolome and insulin sensitivity. Nature.

[72] Wu X., Liu X., Tan H., Song J., Ma S., Tan Y. (2025). Longitudinal change and causal relationship between gut microbiota and gestational diabetes mellitus. Diabetol. Metab. Syndr..

[73] De Vadder F., Kovatcheva-Datchary P., Zitoun C., Duchampt A., Bäckhed F., Mithieux G. (2016). Microbiota-produced succinate improves glucose homeostasis via intestinal gluconeogenesis. Cell Metab..

[74] Chung W.S.F., Walker A.W., Bosscher D., Garcia-Campayo V., Wagner J., Parkhill J., Duncan S.H., Flint H.J. (2020). Relative abundance of the Prevotella genus within the human gut microbiota of elderly volunteers determines the inter-individual responses to dietary supplementation with wheat bran arabinoxylan-oligosaccharides. BMC Microbiol..

[75] Larsen J.M. (2017). The immune response to Prevotella bacteria in chronic inflammatory disease. Immunology.

[76] Prasoodanan P.K.V., Sharma A.K., Mahajan S., Dhakan D.B., Maji A., Scaria J., Sharma V.K. (2021). Western and non-western gut microbiomes reveal new roles of Prevotella in carbohydrate metabolism and mouth–gut axis. npj Biofilms Microbiomes.

[77] De Filippis F., Pellegrini N., Laghi L., Gobbetti M., Ercolini D. (2016). Unusual sub-genus associations of faecal *Prevotella* and *Bacteroides* with specific dietary patterns. Microbiome.

[78] Thingholm L.B., Bang C., Rühlemann M.C., Starke A., Sicks F., Kaspari V., Jandowsky A., Frölich K., Ismer G., Bernhard A. (2021). Ecology impacts the decrease of Spirochaetes and *Prevotella* in the fecal gut microbiota of urban humans. BMC Microbiol..

[79] Gellman R.H., Olm M.R., Terrapon N., Enam F., Higginbottom S.K., Sonnenburg J.L., Sonnenburg E.D. (2023). Hadza Prevotella require diet-derived microbiota-accessible carbohydrates to persist in mice. Cell Rep..

[80] Yang C., Lan R., Zhao L., Pu J., Hu D., Yang J., Zhou H., Han L., Ye L., Jin D. (2024). Prevotella copri alleviates hyperglycemia and regulates gut microbiota and metabolic profiles in mice. Msystems.

[81] Péan N., Le Lay A., Brial F., Wasserscheid J., Rouch C., Vincent M., Myridakis A., Hedjazi L., Dumas M.-E., Grundberg E. (2020). Dominant gut Prevotella copri in gastrectomised non-obese diabetic Goto–Kakizaki rats improves glucose homeostasis through enhanced FXR signalling. Diabetologia.

[82] Verbrugghe P., Brynjólfsson J., Jing X., Björck I., Hållenius F., Nilsson A. (2021). Evaluation of hypoglycemic effect, safety and immunomodulation of Prevotella copri in mice. Sci. Rep..

[83] Eerola E., Möttönen T., Hannonen P., Luukkainen R., Kantola I., Vuori K., Tuominen J., Toivanen P. (1994). Intestinal flora in early rheumatoid arthritis. Rheumatology.

[84] Zhang X., Rimpiläinen M., Šimelyte E., Toivanen P. (2001). Characterisation of Eubacterium cell wall: Peptidoglycan structure determines arthritogenicity. Ann. Rheum. Dis..

[85] Shehata F., Dwyer K.M., Axtens M., McGee S.L., Rivera L.R. (2025). Impact of a Lifestyle Intervention on Gut Microbiome Composition: A Quasi-Controlled Before-and-After Analysis. Metabolites.

[86] An J., Kwon H., Oh S.-Y., Kim Y.J. (2025). Association between breast cancer risk factors and blood microbiome in patients with breast cancer. Sci. Rep..

[87] Zhao S., Lau R., Zhong Y., Chen M.H. (2024). Lactate cross-feeding between *Bifidobacterium* species and *Megasphaera indica* contributes to butyrate formation in the human colonic environment. Appl. Environ. Microbiol..

[88] Carrizales-Sánchez A.K., Tamez-Rivera O., Rodríguez-Gutiérrez N.A., Elizondo-Montemayor L., Gradilla-Hernández M.S., García-Rivas G., Pacheco A., Senés-Guerrero C. (2023). Characterization of gut microbiota associated with metabolic syndrome and type-2 diabetes mellitus in Mexican pediatric subjects. BMC Pediatr..

[89] Sun J., Ma X., Yang L., Jin X., Zhao M., Xi B., Song S. (2023). The number of metabolic syndrome risk factors predicts alterations in gut microbiota in Chinese children from the Huantai study. BMC Pediatr..

[90] Wang J., Zhuang P., Lin B., Zheng J., Li H., Tang W., Ye W., Chen X., Zheng M. (2024). Comparative analysis of gut microbiota in metabolic syndrome and obese children from Southeastern China. Front. Microbiol..

[91] Gallardo-Becerra L., Cornejo-Granados F., García-López R., Valdez-Lara A., Bikel S., Canizales-Quinteros S., López-Contreras B.E., Mendoza-Vargas A., Nielsen H., Ochoa-Leyva A. (2020). Metatranscriptomic analysis to define the Secrebiome, and 16S rRNA profiling of the gut microbiome in obesity and metabolic syndrome of Mexican children. Microb. Cell Fact..

[92] Xiao R., Chen Y., Zhu X., Wang L., Tian P., Jin X., Liang M., Chen Z., Zhang T., Qian L. (2024). A randomised double-blind placebo-controlled trial of a probiotic combination for manipulating the gut microbiota and managing metabolic syndrome. Food Biosci..

[93] Li Y., Su X., Zhang L., Liu Y., Shi M., Lv C., Gao Y., Xu D., Wang Z. (2019). Dysbiosis of the gut microbiome is associated with CKD5 and correlated with clinical indices of the disease: A case–controlled study. J. Transl. Med..

[94] Feng C., Yang M., Yang Z., Liao X., Jiang S., Li L., Lin H., Sun Y., Wei Z., Weng Z. (2026). Longitudinal Interaction Between Individualized Gut Microbial Dynamics and Diet Is Associated with Metabolic Health in School-Aged Children. Nutrients.

[95] Liu T., Petersen C., Zhao N., Moraes T.J., Subbarao P., Simons E., Azad M.B., Miliku K., Bode L., Moore B. (2026). Human Milk and Infant Gut Microbiome in Association With Infant Fecal Metabolome and Child Blood Pressure. JAMA Netw. Open.

[96] Stewart C.J., Ajami N.J., O’Brien J.L., Hutchinson D.S., Smith D.P., Wong M.C., Ross M.C., Lloyd R.E., Doddapaneni H., Metcalf G.A. (2018). Temporal development of the gut microbiome in early childhood from the TEDDY study. Nature.

[97] Barone M., Ramayo-Caldas Y., Estellé J., Tambosco K., Chadi S., Maillard F., Gallopin M., Planchais J., Chain F., Kropp C. (2023). Gut barrier-microbiota imbalances in early life lead to higher sensitivity to inflammation in a murine model of C-section delivery. Microbiome.

[98] Grant E.T., Boudaud M., Muller A., Macpherson A.J., Desai M.S. (2023). Maternal diet and gut microbiome composition modulate early-life immune development. EMBO Mol. Med..

[99] Suriano F., Vieira-Silva S., Falony G., de Wouters d’Oplinter A., Paone P., Delzenne N.M., Everard A., Raes J., Van Hul M., Cani P.D. (2023). Fat and not sugar as the determining factor for gut microbiota changes, obesity, and related metabolic disorders in mice. Am. J. Physiol.-Endocrinol. Metab..

[100] Guo K., Figueroa-Romero C., Noureldein M., Hinder L.M., Sakowski S.A., Rumora A.E., Petit H., Savelieff M.G., Hur J., Feldman E.L. (2023). Gut microbiota in a mouse model of obesity and peripheral neuropathy associated with plasma and nerve lipidomics and nerve transcriptomics. Microbiome.

[101] Jeon C.W., Lee H.Y., Kim H.S., Seo M.J., Park K.W., Yoon J.-H. (2025). Anti-Obesity Effects and Changes of Fecal Microbiome by Lactic Acid Bacteria from Grains in a High-Fat Diet Mouse Model. Int. J. Mol. Sci..

[102] Qiu Y., Wu L., Zhou W., Wang F., Li N., Wang H., He R., Tian Y., Liu Z. (2024). Day and night reversed feeding aggravates high-fat diet-induced abnormalities in intestinal flora and lipid metabolism in adipose tissue of mice. J. Nutr..

[103] Chen D., Wang A., Lv J., Peng Y., Zheng Y., Zuo J., Kan J., Zong S., Zeng X., Liu J. (2024). Tea (*Camellia sinensis* L.) flower polysaccharide attenuates metabolic syndrome in high-fat diet induced mice in association with modulation of gut microbiota. Int. J. Biol. Macromol..

[104] Song Q., Cheng S.W., Zou J., Li K.S.L., Cheng H., Lau D.T.W., Han Q., Yang X., Shaw P.C., Zuo Z. (2024). Role of gut microbiota on regulation potential of Dendrobium officinale Kimura & Migo in metabolic syndrome: In-vitro fermentation screening and in-vivo verification in db/db mice. J. Ethnopharmacol..

[105] Cao H., Xu J., Wang H., Yi W., Yang D., Yang J., Sun J., Wang Y., Zhang F., Yan J. (2025). Fecal microbiota transplantation mitigates postdieting weight regain in mice by modulating the gut-liver axis. BMC Microbiol..

[106] Huang S., Dong S., Lin L., Ma Q., Xu M., Ni L., Fan Q. (2023). Inulin ameliorates metabolic syndrome in high-fat diet-fed mice by regulating gut microbiota and bile acid excretion. Front. Pharmacol..

[107] Wu H., Wang X., Fang X., Lian F., Li M., Liao J., Dai D., Tian J. (2022). Metformin modulates the gut microbiome in a mice model of high-fat diet-induced glycolipid metabolism disorder. BMJ Open Diabetes Res. Care.

[108] Elie C., Perret M., Hage H., Sentausa E., Hesketh A., Louis K., Fritah-Lafont A., Leissner P., Vachon C., Rostaing H. (2023). Comparison of DNA extraction methods for 16S rRNA gene sequencing in the analysis of the human gut microbiome. Sci. Rep..

[109] Rintala A., Pietilä S., Munukka E., Eerola E., Pursiheimo J.-P., Laiho A., Pekkala S., Huovinen P. (2017). Gut microbiota analysis results are highly dependent on the 16S rRNA gene target region, whereas the impact of DNA extraction is minor. J. Biomol. Tech. JBT.

[110] Almeida A., Mitchell A.L., Tarkowska A., Finn R.D. (2018). Benchmarking taxonomic assignments based on 16S rRNA gene profiling of the microbiota from commonly sampled environments. Gigascience.

[111] Zaheer R., Noyes N., Ortega Polo R., Cook S.R., Marinier E., Van Domselaar G., Belk K.E., Morley P.S., McAllister T.A. (2018). Impact of sequencing depth on the characterization of the microbiome and resistome. Sci. Rep..

[112] Joos L., Beirinckx S., Haegeman A., Debode J., Vandecasteele B., Baeyen S., Goormachtig S., Clement L., De Tender C. (2020). Daring to be differential: Metabarcoding analysis of soil and plant-related microbial communities using amplicon sequence variants and operational taxonomical units. BMC Genom..

[113] Deschasaux M., Bouter K.E., Prodan A., Levin E., Groen A.K., Herrema H., Tremaroli V., Bakker G.J., Attaye I., Pinto-Sietsma S.-J. (2018). Depicting the composition of gut microbiota in a population with varied ethnic origins but shared geography. Nat. Med..

[114] Jardon K.M., Umanets A., Gijbels A., Trouwborst I., Hul G.B., Siebelink E., Vliex L.M.M., Bastings J.J.A.J., Argamasilla R., Chenal E. (2025). Distinct gut microbiota and metabolome features of tissue-specific insulin resistance in overweight and obesity. Gut Microbes.

[115] Wang P.X., Deng X.R., Zhang C.H., Yuan H.J. (2020). Gut microbiota and metabolic syndrome. Chin. Med. J..

[116] Brown A.C., Valiere A. (2004). Probiotics and medical nutrition therapy. Nutr. Clin. Care Off. Publ. Tufts Univ..

[117] Schachtsiek M., Hammes W.P., Hertel C. (2004). Characterization of *Lactobacillus coryniformis* DSM 20001T surface protein Cpf mediating coaggregation with and aggregation among pathogens. Appl. Environ. Microbiol..

[118] Wang Y., Dilidaxi D., Wu Y., Sailike J., Sun X., Nabi X.-h. (2020). Composite probiotics alleviate type 2 diabetes by regulating intestinal microbiota and inducing GLP-1 secretion in db/db mice. Biomed. Pharmacother..

[119] Tuohy K.M., Probert H.M., Smejkal C.W., Gibson G.R. (2003). Using probiotics and prebiotics to improve gut health. Drug Discov. Today.

[120] Simon O., Vahjen W., Scharek L. (2005). Micro-organisms as feed additives-probiotics. Adv. Pork Prod..

[121] Depommier C., Everard A., Druart C., Plovier H., Van Hul M., Vieira-Silva S., Falony G., Raes J., Maiter D., Delzenne N.M. (2019). Supplementation with *Akkermansia muciniphila* in overweight and obese human volunteers: A proof-of-concept exploratory study. Nat. Med..

[122] Zhang Y., Liu R., Chen Y., Cao Z., Liu C., Bao R., Wang Y., Huang S., Pan S., Qin L. (2025). *Akkermansia muciniphila* supplementation in patients with overweight/obese type 2 diabetes: Efficacy depends on its baseline levels in the gut. Cell Metab..

[123] Zhou K. (2017). Strategies to promote abundance of *Akkermansia muciniphila*, an emerging probiotics in the gut, evidence from dietary intervention studies. J. Funct. Foods.

[124] Wang J., Tang H., Zhang C., Zhao Y., Derrien M., Rocher E., van-Hylckama Vlieg J.E.T., Strissel K., Zhao L., Obin M. (2015). Modulation of gut microbiota during probiotic-mediated attenuation of metabolic syndrome in high fat diet-fed mice. ISME J..

[125] Li N., Gong Y., Zhu Y., Li B., Wang C., Wang Z., Wang J., Huang J., Bian J., Zhang Y. (2024). Exogenous acetate attenuates inflammatory responses through HIF-1α-dependent glycolysis regulation in macrophage. Cell. Mol. Life Sci..

[126] Olaniyi K.S., Amusa O.A., Areola E.D., Olatunji L.A. (2020). Suppression of HDAC by sodium acetate rectifies cardiac metabolic disturbance in streptozotocin–nicotinamide-induced diabetic rats. Exp. Biol. Med..

[127] Xu M., Jiang Z., Wang C., Li N., Bo L., Zha Y., Bian J., Zhang Y., Deng X. (2019). Acetate attenuates inflammasome activation through GPR43-mediated Ca^2+^-dependent NLRP3 ubiquitination. Exp. Mol. Med..

[128] Chen J.J., Wang R., Li X.-f., Wang R.-l. (2011). *Bifidobacterium longum* supplementation improved high-fat-fed-induced metabolic syndrome and promoted intestinal Reg I gene expression. Exp. Biol. Med..

[129] Chen J., Wang R., Li X.-F., Wang R.-L. (2012). *Bifidobacterium adolescentis* supplementation ameliorates visceral fat accumulation and insulin sensitivity in an experimental model of the metabolic syndrome. Br. J. Nutr..

[130] Tenorio-Jiménez C., Martínez-Ramírez M.J., Del Castillo-Codes I., Arraiza-Irigoyen C., Tercero-Lozano M., Camacho J., Chueca N., García F., Olza J., Plaza-Díaz J. (2019). Lactobacillus reuteri V3401 reduces inflammatory biomarkers and modifies the gastrointestinal microbiome in adults with metabolic syndrome: The PROSIR study. Nutrients.

[131] Dao M.C., Everard A., Aron-Wisnewsky J., Sokolovska N., Prifti E., Verger E.O., Kayser B.D., Levenez F., Chilloux J., Hoyles L. (2016). *Akkermansia muciniphila* and improved metabolic health during a dietary intervention in obesity: Relationship with gut microbiome richness and ecology. Gut.

[132] Everard A., Belzer C., Geurts L., Ouwerkerk J.P., Druart C., Bindels L.B., Guiot Y., Derrien M., Muccioli G.G., Delzenne N.M. (2013). Cross-talk between *Akkermansia muciniphila* and intestinal epithelium controls diet-induced obesity. Proc. Natl. Acad. Sci. USA.

[133] Le Chatelier E., Nielsen T., Qin J., Prifti E., Hildebrand F., Falony G., Almeida M., Arumugam M., Batto J.-M., Kennedy S. (2013). Richness of human gut microbiome correlates with metabolic markers. Nature.

[134] Bernini L.J., Simão A.N.C., Alfieri D.F., Lozovoy M.A.B., Mari N.L., de Souza C.H.B., Dichi I., Costa G.N. (2016). Beneficial effects of Bifidobacterium lactis on lipid profile and cytokines in patients with metabolic syndrome: A randomized trial. Effects of probiotics on metabolic syndrome. Nutrition.

[135] Bernini L.J., Simão A.N.C., de Souza C.H., Alfieri D.F., Segura L.G., Costa G.N., Dichi I. (2018). Effect of Bifidobacterium lactis HN019 on inflammatory markers and oxidative stress in subjects with and without the metabolic syndrome. Br. J. Nutr..

[136] Numnark D., Klaisung O., Panprame P., Plubcharoensook P. (2025). Effect of Bifidobacterium Breve on Lipid Profile and Body Fat Reduction in Patients with Metabolic Syndrome: A Randomized, Double-Blind, Placebo-Controlled, Clinical Trial. J. Curr. Sci. Technol..

[137] Gibson G.R., Roberfroid M.B. (1995). Dietary modulation of the human colonic microbiota: Introducing the concept of prebiotics. J. Nutr..

[138] Baba Y., Tsuge D., Aoki R. (2025). Enhancement of carbohydrate metabolism by probiotic and prebiotic intake promotes short-chain fatty acid production in the gut microbiome: A randomized, double-blind, placebo-controlled crossover trial. Biosci. Biotechnol. Biochem..

[139] Gargari B.P., Dehghan P., Aliasgharzadeh A., Jafar-Abadi M.A. (2013). Effects of high performance inulin supplementation on glycemic control and antioxidant status in women with type 2 diabetes. Diabetes Metab. J..

[140] Niness K.R. (1999). Inulin and oligofructose: What are they?. J. Nutr..

[141] Tomasik P.J., Tomasik P. (2003). Probiotics and prebiotics. Cereal Chem..

[142] Jacobs J.P., Lee M.L., Rechtman D.J., Sun A.K., Autran C., Niklas V. (2023). Human milk oligosaccharides modulate the intestinal microbiome of healthy adults. Sci. Rep..

[143] Natale A., Fiori F., Turati F., La Vecchia C., Parpinel M., Rossi M. (2025). Quantification of Naturally Occurring Prebiotics in Selected Foods. Nutrients.

[144] Salli K., Hirvonen J., Siitonen J., Ahonen I., Anglenius H., Maukonen J. (2020). Selective utilization of the human milk oligosaccharides 2′-fucosyllactose, 3-fucosyllactose, and difucosyllactose by various probiotic and pathogenic bacteria. J. Agric. Food Chem..

[145] Rastall R.A., Maitin V. (2002). Prebiotics and synbiotics: Towards the next generation. Curr. Opin. Biotechnol..

[146] Roberfroid M., Gibson G.R., Hoyles L., McCartney A.L., Rastall R., Rowland I., Wolvers D., Watzl B., Szajewska H., Stahl B. (2010). Prebiotic effects: Metabolic and health benefits. Br. J. Nutr..

[147] Van Loo J., Coussement P., De Leenheer L., Hoebregs H., Smits G. (1995). On the presence of inulin and oligofructose as natural ingredients in the western diet. Crit. Rev. Food Sci. Nutr..

[148] Delzenne N.M., Neyrinck A.M., Cani P.D. (2013). Gut microbiota and metabolic disorders: How prebiotic can work?. Br. J. Nutr..

[149] Cani P.D., Neyrinck A.M., Fava F., Knauf C., Burcelin R.G., Tuohy K.M., Gibson G.R., Delzenne N.M. (2007). Selective increases of bifidobacteria in gut microflora improve high-fat-diet-induced diabetes in mice through a mechanism associated with endotoxaemia. Diabetologia.

[150] Kumar S.A., Ward L.C., Brown L. (2016). Inulin oligofructose attenuates metabolic syndrome in high-carbohydrate, high-fat diet-fed rats. Br. J. Nutr..

[151] Komatsu Y., Aoyama K., Yoneda M., Ashikawa S., Nakano S., Kawai Y., Cui X., Furukawa N., Ikeda K., Nagata K. (2021). The prebiotic fiber inulin ameliorates cardiac, adipose tissue, and hepatic pathology, but exacerbates hypertriglyceridemia in rats with metabolic syndrome. Am. J. Physiol.-Heart Circ. Physiol..

[152] Tian R., Hong J., Zhao J., Zhou D., Liu Y., Jiao Z., Song J., Zhang Y., Meng L., Yu M. (2022). Overall structural alteration of gut microbiota and relationships with risk factors in patients with metabolic syndrome treated with inulin alone and with other agents: An open-label pilot study. Mediat. Inflamm..

[153] Hall C.V., Hepsomali P., Dalile B., Scapozza L., Gurry T. (2024). Effects of a diverse prebiotic fibre blend on inflammation, the gut microbiota and affective symptoms in metabolic syndrome: A pilot open-label randomised controlled trial. Br. J. Nutr..

[154] Jiang H., Cai M., Shen B., Wang Q., Zhang T., Zhou X. (2022). Synbiotics and gut microbiota: New perspectives in the treatment of type 2 diabetes mellitus. Foods.

[155] Wang L., Yang H., Huang H., Zhang C., Zuo H.-X., Xu P., Niu Y.-M., Wu S.-S. (2019). Inulin-type fructans supplementation improves glycemic control for the prediabetes and type 2 diabetes populations: Results from a GRADE-assessed systematic review and dose–response meta-analysis of 33 randomized controlled trials. J. Transl. Med..

[156] Lauw S., Kei N., Chan P.L., Yau T.K., Ma K.L., Szeto C.Y.Y., Lin J.S.-C., Wong S.H., Cheung P.C.K., Kwan H.S. (2023). Effects of synbiotic supplementation on metabolic syndrome traits and gut microbial profile among overweight and obese Hong Kong Chinese individuals: A randomized trial. Nutrients.

[157] Li N., Zhu Z., Wu S., Gong D., Day R., Vijayakumar V., Yu X., Chen Q., Feng Y., Wang Q. (2025). Effects of a novel synbiotic intervention on abdominal visceral fat reductions and gut microbiota in overweight and obese adults: A randomized, double-blind, placebo-controlled trial. Clin. Nutr..

[158] Zolghadrpour M.-A., Jowshan M.-R., Heidari Seyedmahalleh M., Karimpour F., Imani H., Asghari S. (2024). The effect of a new developed synbiotic yogurt consumption on metabolic syndrome components in adults with metabolic syndrome: A randomized controlled clinical trial. Nutr. Diabetes.

[159] Eslamparast T., Zamani F., Hekmatdoost A., Sharafkhah M., Eghtesad S., Malekzadeh R., Poustchi H. (2014). Effects of synbiotic supplementation on insulin resistance in subjects with the metabolic syndrome: A randomised, double-blind, placebo-controlled pilot study. Br. J. Nutr..

[160] Rabiei S., Hedayati M., Rashidkhani B., Saadat N., Shakerhossini R. (2019). The effects of synbiotic supplementation on body mass index, metabolic and inflammatory biomarkers, and appetite in patients with metabolic syndrome: A triple-blind randomized controlled trial. J. Diet. Suppl..

[161] Cicero A.F.G., Fogacci F., Bove M., Giovannini M., Borghi C. (2021). Impact of a short-term synbiotic supplementation on metabolic syndrome and systemic inflammation in elderly patients: A randomized placebo-controlled clinical trial. Eur. J. Nutr..

[162] Aguilar-Toalá J., Garcia-Varela R., Garcia H., Mata-Haro V., González-Córdova A., Vallejo-Cordoba B., Hernández-Mendoza A. (2018). Postbiotics: An evolving term within the functional foods field. Trends Food Sci. Technol..

[163] Salminen S., Collado M.C., Endo A., Hill C., Lebeer S., Quigley E.M., Sanders M.E., Shamir R., Swann J.R., Szajewska H. (2021). The International Scientific Association of Probiotics and Prebiotics (ISAPP) consensus statement on the definition and scope of postbiotics. Nat. Rev. Gastroenterol. Hepatol..

[164] Xiao S.-D., De Zhang Z., Lu H., Jiang S.H., Liu H.Y., Wang G.S., Xu G.M., Zhang Z.B., Lin G.J., Wang G.L. (2003). Multicenter, randomized, controlled trial of heat-killed Lactobacillus acidophilus LB in patients with chronic diarrhea. Adv. Ther..

[165] Andresen V., Gschossmann J., Layer P. (2020). Heat-inactivated *Bifidobacterium bifidum* MIMBb75 (SYN-HI-001) in the treatment of irritable bowel syndrome: A multicentre, randomised, double-blind, placebo-controlled clinical trial. Lancet Gastroenterol. Hepatol..

[166] Vinderola G., Sanders M.E., Salminen S. (2022). The concept of postbiotics. Foods.

[167] Scott E., De Paepe K., Van de Wiele T. (2022). Postbiotics and their health modulatory biomolecules. Biomolecules.

[168] Štofilová J., Kvaková M., Kamlárová A., Hijová E., Bertková I., Guľašová Z. (2022). Probiotic-based intervention in the treatment of ulcerative colitis: Conventional and new approaches. Biomedicines.

[169] Ríus A., Kaufman J., Li M., Hanigan M.D., Ipharraguerre I. (2022). Physiological responses of Holstein calves to heat stress and dietary supplementation with a postbiotic from *Aspergillus oryzae*. Sci. Rep..

[170] Liu Y., Wang J., Wu C. (2022). Modulation of gut microbiota and immune system by probiotics, pre-biotics, and post-biotics. Front. Nutr..

[171] Chu C., Jiang J., Yu L., Li Y., Zhang S., Zhou W., Wang Q., Zhao J., Zhai Q., Tian F. (2023). *Bifidobacterium longum* CCFM1077 attenuates hyperlipidemia by modulating the gut microbiota composition and fecal metabolites: A randomized, double-blind, placebo-controlled clinical trial. Engineering.

[172] Uchinaka A., Azuma N., Mizumoto H., Nakano S., Minamiya M., Yoneda M., Aoyama K., Komatsu Y., Yamada Y., Murohara T. (2018). Anti-inflammatory effects of heat-killed Lactobacillus plantarum L-137 on cardiac and adipose tissue in rats with metabolic syndrome. Sci. Rep..

[173] Bouter K.E., Bakker G., Levin E., Hartstra A., Kootte R., Udayappan S., Katiraei S., Bahler L., Gilijamse P., Tremaroli V. (2018). Differential metabolic effects of oral butyrate treatment in lean versus metabolic syndrome subjects. Clin. Transl. Gastroenterol..

[174] Allegretti J.R., Mullish B.H., Kelly C., Fischer M. (2019). The evolution of the use of faecal microbiota transplantation and emerging therapeutic indications. Lancet.

[175] Zuppi M., Vatanen T., Wilson B.C., Golovina E., Portlock T., Cutfield W.S., Vickers M.H., O’Sullivan J.M. (2024). Fecal microbiota transplantation alters gut phage communities in a clinical trial for obesity. Microbiome.

[176] Vrieze A., Van Nood E., Holleman F., Salojärvi J., Kootte R.S., Bartelsman J.F.W.M., Dallinga-Thie G.M., Ackermans M.T., Serlie M.J., Oozeer R. (2012). Transfer of intestinal microbiota from lean donors increases insulin sensitivity in individuals with metabolic syndrome. Gastroenterology.

[177] Kootte R.S., Levin E., Salojärvi J., Smits L.P., Hartstra A.V., Udayappan S.D., Hermes G., Bouter K.E., Koopen A.M., Holst J.J. (2017). Improvement of insulin sensitivity after lean donor feces in metabolic syndrome is driven by baseline intestinal microbiota composition. Cell Metab..

[178] Yunes R.A., Poluektova E.U., Dyachkova M.S., Klimina K.M., Kovtun A.S., Averina O.V., Orlova V.S., Danilenko V.N. (2016). GABA production and structure of gadB/gadC genes in Lactobacillus and Bifidobacterium strains from human microbiota. Anaerobe.

[179] Marques T.M., Patterson E., Wall R., O’Sullivan O., Fitzgerald G.F., Cotter P.D., Dinan T.G., Cryan J.F., Ross R.P., Stanton C. (2016). Influence of GABA and GABA-producing *Lactobacillus brevis* DPC 6108 on the development of diabetes in a streptozotocin rat model. Benef. Microbes.

[180] Tian J., Dang H.N., Yong J., Chui W.-S., Dizon M.P.G., Yaw C.K.Y., Kaufman D.L. (2011). Oral treatment with γ-aminobutyric acid improves glucose tolerance and insulin sensitivity by inhibiting inflammation in high fat diet-fed mice. PLoS ONE.

[181] Udayappan S., Manneras-Holm L., Chaplin-Scott A., Belzer C., Herrema H., Dallinga-Thie G.M., Duncan S.H., Stroes E.S.G., Groen A.K., Flint H.J. (2016). Oral treatment with *Eubacterium hallii* improves insulin sensitivity in db/db mice. npj Biofilms Microbiomes.

[182] Boey D., Sainsbury A., Herzog H. (2007). The role of peptide YY in regulating glucose homeostasis. Peptides.

[183] Farilla L., Bulotta A., Hirshberg B., Li Calzi S., Khoury N., Noushmehr H., Bertolotto C., Di Mario U., Harlan D.M., Perfetti R. (2003). Glucagon-Like Peptide 1 Inhibits Cell Apoptosis and Improves Glucose Responsiveness of Freshly Isolated Human Islets. Endocrinology.

[184] MacDonald P.E., El-kholy W., Riedel M.J., Salapatek A.M.F., Light P.E., Wheeler M.B. (2002). The Multiple Actions of GLP-1 on the Process of Glucose-Stimulated Insulin Secretion. Diabetes.

